# A neurologist and ataxia: using eye movements to learn about the cerebellum

**DOI:** 10.1186/s40673-018-0081-2

**Published:** 2018-02-07

**Authors:** David S. Zee

**Affiliations:** Departments of Neurology, Ophthalamology, Otolaryngology-Head and Neck Surgery, and Neuroscience, The Johns Hopkins University School of Medicine, The Johns Hopkins Hospital, Path 2-210, Baltimore, MD 21287 USA

**Keywords:** Cerebellum, Ataxia, Eye movements

## Abstract

The cerebellum, its normal functions and its diseases, and especially its relation to the control of eye movements, has been at the heart of my academic career. Here I review how this came about, with an emphasis on epiphanies, “tipping points” and the influences of mentors, colleagues and trainees. I set a path for young academicians, both clinicians and basic scientists, with some guidelines for developing a productive and rewarding career in neuroscience.

**Electronic supplementary material:**

The online version of this article (10.1186/s40673-018-0081-2) contains supplementary material, which is available to authorized users.

## Background

The normal functions of the cerebellum and its diseases has been at the heart of my academic career of more than 45 years – both in clinical care of patients, and in clinical and experimental research. More than 85 of my publications have the word cerebellum in the title, or the cerebellum is central to the problems discussed in the publication (see Additional files 1 and 2). Most of these publications emphasize some aspect of the relationship of the cerebellum to the control of eye movements, including all its subtypes, vestibular, saccade, pursuit and vergence. The visual symptoms from the disturbed ocular motor control in cerebellar patients are often extremely disabling and life altering, for example, double vision due to ocular misalignment, and oscillopsia due to nystagmus or other unwanted ocular oscillations. This has been one reason for my sustained focus, over so many years, on this relatively small, but vital part of the brain. My interests in the cerebellum followed upon a series of *epiphanies*, based on people – patients, physicians and scientists – with whom I came in contact; on the times; and on chance and good fortune. At every step of the way, I reached a “*tipping point”* that pushed me in a new direction or to a particular person who became an influential mentor, colleague, or trainee. Here I recapitulate some of this story and based on my experience suggest some “tips” for success (Table 1), which I hope will help those early in their careers as they make decisions about how their academic lives are to unfold.

**Table 1:** Ten Tips For Academic Success

• Keep an eye out for something new, exciting, and important to study.
• Interact and collaborate with colleagues and trainees who have skills you do not or see or do things differently than you. Look for analogies to see how problems have been solved in other fields.
• Listen, more than talk.
• Pick a mentor who, at any level of career, is looking to the future and striving to be at the forefront of the field. Joining a new enterprise at its inception under a young, inspiring leader, is often as good or even better option than joining a large, established enterprise under an older but busy entrenched leader.
• Know, but not necessarily accept, what has been said, written and accomplished in the past.
• Persevere but be willing to change course when you should change course; be focused but flexible.
• Make your research quantitative and hypothesis driven, and when things look like they fit, try to prove your hypotheses wrong!
• Learn how to teach effectively and how to write concisely. Get feedback from mentors and students.
• Broaden your horizons. Meet colleagues and students from other countries and cultures. You gain much from collaborating, teaching and learning with them, and establishing enduring friendships. Take sabbaticals.
• If you are a clinician, learn from your patients, take physiology and anatomy to the bedside, figure out how the brain works and write papers, or even a book to educate your colleagues.

### Why I chose neuroscience

“Keep an eye out for something new and exciting to study”. In 1965, I began medical school classwork at Johns Hopkins in neuroanatomy and immediately became enthralled with the brain, marveling at its exquisite connectivity. Later in the same year, I was able to watch Professor Vernon Mountcastle, chair of physiology and an eminent neurophysiologist especially for his discovery of the columnar architecture of somatosensory cerebral cortex, perform experiments in his laboratory. He was recording the activity in individual nerve fibers of experimental animals in response to different sensory stimuli. The ability to “see” how neural activity in the brain encodes experiences from the outside world was an epiphany for me and further piqued my interest in a career in neuroscience. In 1966, after my first year of medical school, I opted for a summer elective with the chair of the anatomy department, Professor David Bodian, well known for his seminal studies on the pathogenesis of poliomyelitis, which made possible the development of the polio vaccine. He also developed the “Bodian” silver stain for identifying nerve fibers and nerve endings in neuroanatomical sections. That summer we spent many hours together at the microscope, examining the upper cervical spinal cord trying to decipher propriospinal pathways. Nowadays how often does a departmental chair have even a small amount of time, let alone almost daily sessions, to spend with a first year medical student on an elective in the laboratory? My fascination with the anatomical and physiological organization of the brain continued throughout medical school so that on our own time, a classmate, Tom Woolsey, who was in a similar state of anatomical “ecstasy” and I dissected a gross brain specimen. We were trying to envision in three dimensions the complicated relationships among the fluid spaces and fissures of the brain. Tom ultimately achieved considerable fame for his discovery, while still a medical student, of the “barrel” organization of the projections of the whiskers (vibrissa) in the cerebral cortex of the rat.

### Why I chose neurology

When it came time to choose a clinical specialty, neurology was the natural choice. Again, an experience (another summer elective, this time at the Mayo Clinic in neurology in 1968), and an exposure to some of the giants of clinical neurology there (Dr. Frank Howard of myasthenia gravis fame, and Drs. Thomas Kearns and Robert Hollenhorst of neuroophthalmolgy fame) made neurology an inevitable decision. My interests in the cerebellum were also stirred at the Mayo Clinic when one of the patients assigned to me was being evaluated for a chronic cerebellar ataxia. I was told to look up a classic paper on cerebellar degeneration in alcoholics by Maurice Victor and colleagues entitled “A restricted form of cerebellar cortical degeneration occurring in alcoholic patients”, which was 109 pages long [1]. I confess I did not read this paper from start to finish but the ability to correlate function and anatomy using the clinical examination and subsequent pathology was a “tipping point” that pushed me towards neurology and eventually the cerebellum. This experience also emphasized to me the importance of reading and knowing the medical literature. “Know, but not necessarily accept, what has been said, written and accomplished in the past.”

### Why I chose neuroophthalmology

All medical students visiting the Mayo Clinic for the summer elective program were obliged to take a week of neuroophthalmology. At that time, I came across the classic textbook, “The Neurology of the Ocular Muscles” by David Cogan, the eminent neuroophthalmologist and Chair of Ophthalmology at the Harvard Medical School. About 6 years later, in 1974-75, while I was serving in the Public Health Service at the National Institutes of Health in Bethesda, by chance my small cubicle was next door to the office of Dr. Cogan. He had relocated to the National Eye Institute in Bethesda after retiring from Harvard. Dr. Cogan took me under his wing and sent me to my first international conference (in Stockholm in 1975) simply as an observer because he thought it would be “good for me”. The other major individual who stirred my interest in neuroophthalmology was Dr. Frank Walsh at Johns Hopkins. As a neurology resident at Hopkins (1970-1973), I attended Dr. Walsh’s Saturday morning neuroophthalmology conferences and he, like Dr. Cogan, took considerable interest in my career. He sent me to an international colloquium on the pupil in Detroit so that I might gain more exposure in the field. Dr. Walsh told me that someone (even a lowly neurology resident) should represent the Wilmer Eye Institute. I have never forgotten the generosity and interest in my early career of these two giants. One important caveat. Take your mentor’s suggestions seriously. Dr. Cogan and I were evaluating a patient with slow saccades and he suggested ocular electromyography might help. He asked if I would be the control subject. I thought he was joking but about 45 min later, I was lying on a table with a huge needle in my lateral rectus (in those days ocular electromyographic needles were large and foreboding). A functional MRI would have shown my entire brain, in some sort of limbic seizure, lighting up as I watched Dr. Cogan approach my eye with needle in hand. I can at least report that the experience was more frightening than painful.

### Why I chose eye movements

Almost every neurology resident at some time during his or her training becomes infatuated with neuroophthalmolgy. Examining the eyes is perhaps the most fascinating part of the neurological evaluation, making the performance of the brain easily accessible to simple visual inspection using only a penlight, ophthalmoscope and a target for the patient to fix upon or track. The findings on the neuroophthalmologic examination are commonly the key to localizing lesions in many parts of the brain and especially in the brainstem and cerebellum. As a second year resident, I attended an introductory lecture for neurology residents on eye movements given by David A. Robinson, a bioengineer and ocular motor physiologist, working in the Wilmer Eye Institute. His topic was the pathophysiology of internuclear ophthalmoplegia (INO), a common ocular motor disorder of the brainstem in which the medial longitudinal fasciculus (MLF), which conveys information to the oculomotor nuclei, is interrupted. He used a simple control systems approach to the signal processing needed to generate normal eye movements, and then derived what happens when there is an interruption in the flow of information in the MLF. This remarkable exposition led to an immediate epiphany. Applying simple mathematics to understanding a complex pattern of pathological eye movements, and being able to pinpoint the location of the defect in the processing of information by the brain, tipped me forever into normal and pathological ocular motor control.

After the lecture, I asked Dave Robinson if I could work with him during my elective time in the last year of my residency. He immediately accepted, saying, “I have been waiting for years for a neurologist to come by and work with me”. Asking Dave Robinson to be my scientific mentor was a key point in my career as he had realized early on how much we could learn about the functioning of the normal brain from examining patients who suffered the unfortunate accidents and diseases of nature. “Pick a mentor who, at any level of career, is looking to the future and striving to be at the forefront of the field”. After I joined his laboratory, we began weekly hospital rounds in which Dave and his graduate students and post-doctoral fellows, and our clinical group, including residents and medical students, would go to the bedside of a patient who had a challenging ocular motor problem. We examined the patient together, and afterwards discussed the mechanism, what new questions to ask, and what experiments could answer them. Publications often grew from these bedside conversations, usually with us challenging Dave to make a model [2]. This experience emphasized for me the importance of interacting with people who came from different fields, with different scientifc and clinical backgrounds and expertise. “Interact and collaborate with colleagues and trainees who have skills you do not or see or do things differently than you”.

**Fig. 1:**
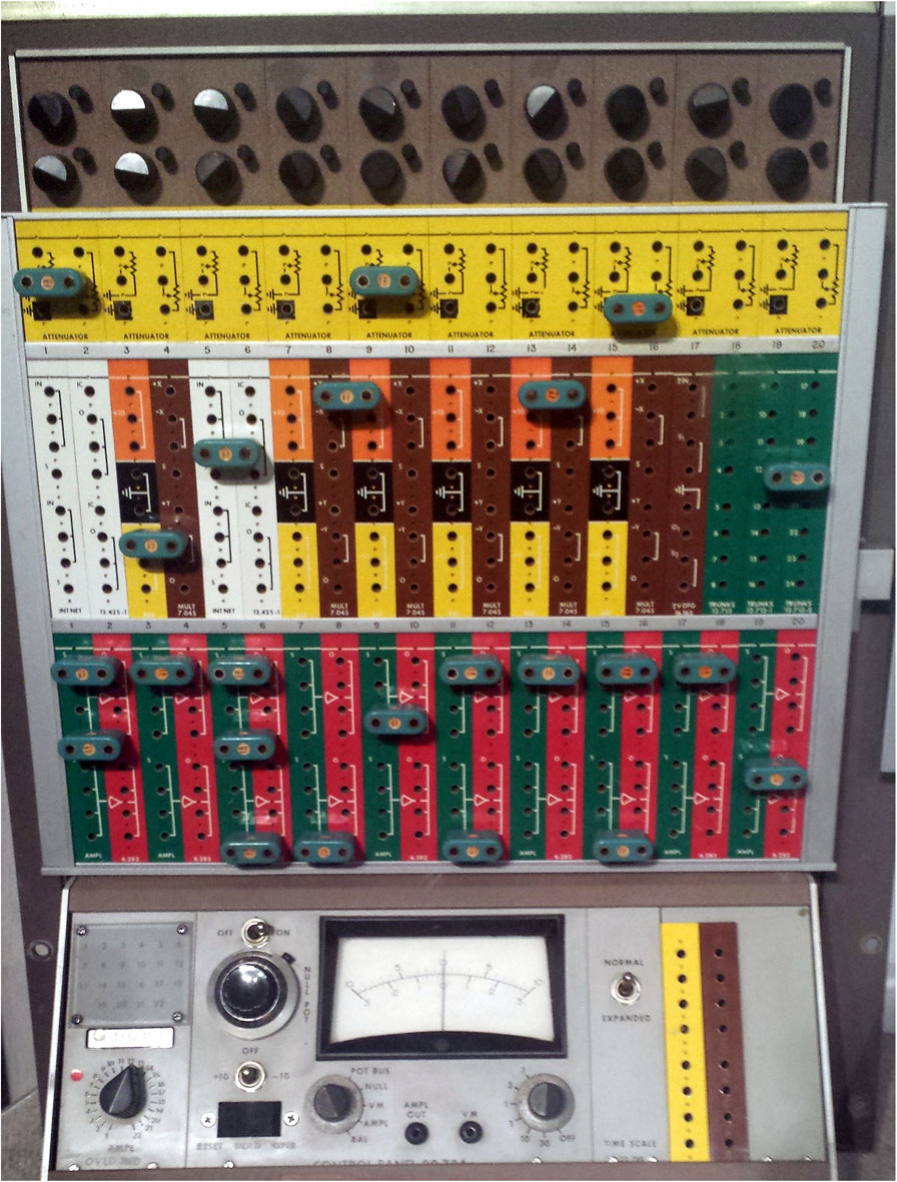
Analog computer in which our first simulations of downbeat nystagmus were made in 1973. Differentiators, integrators and pulse generators were simulated with capacitors, resisters, amplifiers and one-shot multivibrators

When I joined the laboratory in 1972 Dave’s first job was teach me control systems using eye movements as the model. We met several times each week, for an hour or so, one on one. These sessions often involved homework problems for me. Dave and I also sat down at the analog computer together to test our ideas (Fig. 1). This teaching tutorial began with an analysis of signal processing in the vestibulo-ocular reflex (VOR). When the head moves the brain must program an eye movement that is exactly compensatory for us to see clearly when we walk or turn our head. In another epiphany, I realized that understanding the vestibular system – being the fundamental evolutionary scaffolding upon which all the subtypes of eye movements developed – was key for me to becoming an ocular motor clinician-scientist.

The most important projects in Dave’s laboratory at that time related to the function of the cerebellum in the control of the VOR. He was studying how the brain maintains the correct timing (phase) of the VOR, both adaptively in the long-term and in its immediate online control. These experiments led to the idea of a cerebellar ocular motor “repair shop”, compensating when the ocular motor control system goes awry [3]. Another key concept from these experiments emerged that became a fundamental building block in ocular motor physiology – the idea of an ocular motor integrator, not only to insure that the phase of the VOR was correct, but also to hold the eyes still after the eyes finished moving [4]. Gaze-evoked nystagmus, a common sign of cerebellar dysfunction, could then be interpreted as a disorder in a neural network that mathematically integrates a velocity (move) command into a position (holding) command. More recently, this concept of mathematical neural integrators has been applied to the control of the head and other parts of the body by my colleagues Aasef Shaikh and Reza Shadmehr and their collaborators [5, 6]. “Look for analogies to see how problems have been solved in other fields”.

This exciting research in Dave Robinson’s laboratory kindled my interest in both the vestibular system and the cerebellum. Shortly after I started working in the laboratory, the chief of my department, Dr. Guy Mckhann, referred to me several patients with a persistent spontaneous downbeating nystagmus as part of a clinical cerebellar syndrome. Guy McKhann was the new and young chair of a just established department of Neurology at Johns Hopkins. He always looked out for and referred patients with clinical problems that his young trainees might profitably investigate. Guy and I also began a therapeutic drug trial in a group of ataxia patients which was perhaps one of the earliest such trials in cerebellar patients. Unfortunately, the medication was not helpful. Two key paths for my research followed from the investigation of these patients: 1) using control systems models to interpret abnormal eye movements, and 2) developing an animal model in monkeys of the effects of experimental lesions of different parts of the cerebellum on eye movements. First, with Dave Robinson, using the analog computer (Fig. 1) we made a control systems model of downbeat nystagmus. This was one of the first neurological disorders studied and interpreted in this way [7]. This led to my first scientific presentation, at the Association for Research in Vision and Ophthalmology (ARVO) meeting in 1973. Moreover, once we started modeling the disorder we realized we had to know more about the function of the vertical VOR. I realized we could engage and measure the vertical VOR simply by rotating a subject in a vestibular chair around an earth vertical axis with the head tilted 90 degrees to one side to stimulate the vertical semicircular canals. Not a great scientific discovery to be sure, but probably had never been performed on a patient before. The message here, of course, is that mathematical models allow you to test your hypotheses rigorously and suggest new quantitative experiments to challenge your hypotheses. “Make your research quantitative and hypothesis driven, and when things look like they fit, try to prove your hypotheses wrong!” This same approach led to seminal models of the control of saccades and the pathogenesis of various forms of oscillations and nystagmus, which we will discuss later.

### Why I chose the cerebellum

**Fig. 2:**
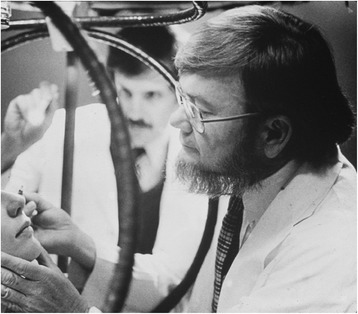
The magnetic field search coil technique applied to human subjects. David A Robinson (right) inserting a small scleral annulus that was developed by Han Collewijn from The Netherlands to precisely measure eye movements, with David Zee (middle) looking on. Circa 1980s

Our studies with downbeat nystagmus pointed to a large gap in knowledge about how the cerebellum works and how cerebellar disease is manifest. The intricate connections of the cerebellum to the brain stem (and now the thalamus and even cerebral cortex) always hung over the question of what is a cerebellar eye sign. “Keep an eye out for something new, exciting, and important to study”. We needed an animal model to study the effects of lesions in the cerebellum on eye movements. With the advent of Robinson’s search coil technique allowing the accurate recording of eye movements, and using monkeys that I could train to fix upon and follow targets, I hoped to make progress toward delineating a cerebellar ocular motor syndrome. Over the next quarter century, we recorded and analyzed eye movements in monkeys before and after focal cerebellar lesions including the flocculus and paraflocculus (tonsil), the dorsal vermis, and the nodulus [8–15]. My long-standing colleagues at Johns Hopkins, Mark Walker, Richard Lewis and Rafael Tamargo played key roles in these experiments. These studies enhanced our clinical diagnostic acumen and our ability to infer what might be the functions of different parts of the cerebellum. At the same time, we carefully quantified eye movements in patients who had naturally occurring dysfunction of the cerebellum and compared their findings with our experimental results (e.g., [16]). We used a version of the search-coil technique for humans to measure eye movements (Fig. 2), and control systems techniques to analyze the data. In a true model of translational research, we went back and forth, iteratively, between studies in patients and in experimental animals, to learn what the cerebellum does and how we might better localize and diagnose lesions of the cerebellum in our patients. We constantly kept in mind the Robinsonian approach; careful measurement, quantitative analysis, hypothesis testing and analytical modelling but always with the patient at the back of our minds, both to improve their lot and to discover what they can teach us about how the brain works.

An exemplar of this approach was developing a model for the premotor circuits that generate saccade commands. We based our ideas on a single patient who made slow saccades as part of a spinocerebellar degeneration [17]. Her saccades were slow because of degeneration in the premotor saccade “burst” neurons within the pontine paramedian reticular formation. Her slow eye movements provided us with the opportunity to see if saccades were preprogrammed and ballistic, as was the conventional wisdom in the 1970s. I reasoned that by jumping the target while a slow saccade was in flight, we could test the idea of preprogramming by seeing if our patient could change the course or direction of her saccades in midflight. Indeed, when the target jumped back to the starting position after she began the saccade, her eyes turned around without stopping and returned to the starting position. If the target jumped onwards in midflight as her eyes began slowing down, her eyes would again pick up speed in response to the new target location and eventually reach the target in just one movement. These results suggested her saccades were under a type of internal feedback control. This “local feedback model” with only slight modifications has stood the test of time for how the brain generates normal saccades. Moreover, this model has been an impetus to many current ideas about how the cerebellum and other structures optimize control of movements, for both immediate online adjustments of motor performance and long-term adaptive motor learning. Furthermore, this model can simulate certain saccadic oscillations such as ocular flutter – intrusive, uncalled for, and often dramatic, back-to-back saccades [18].

Another example of the control systems approach to ocular motor disorders was a study by John Leigh, Dave Robinson, and me of a patient with a cerebellar lesion causing periodic alternating nystagmus (PAN), a disorder in which a spontaneous nystagmus alternates direction every 2 min [19]. It was early on a Saturday morning in the basement of the Wilmer Eye Institute, that Dave, John and I were recording the eye movements of this patient. The idea was to test the then current model of processing of information in the VOR to see how PAN might arise. A key test of the model was how one might stop the nystagmus and John and Dave had come up with some predictions. Accordingly, we measured the patient’s nystagmus that morning and then Dave—working furiously with a paper and pencil— came up with an amplitude and a duration of a rotational vestibular stimulus that if delivered in the right part of the patient’s nystagmus cycle would —the model predicted—stop the nystagmus. We tried it—it worked—and the patient was ecstatic. Her visual blurring from the nystagmus was relieved, albeit for only about 10 min, for the first time in many years! Experiments in animals a few years later showed that a loss of function of Purkinje cells in the cerebellar nodulus was the cause of PAN because of disinhibition and consequent instability of a central “velocity-storage” mechanism within the vestibular nuclei [20].

Fortunately, shortly after we saw our patient, and somewhat serendipitously after a casual discussion with colleagues from the United Kingdom at an ARVO meeting, we reported that baclofen, a GABA-like drug, could permanently stop her nystagmus [21]. Baclofen was a surrogate for the missing GABA-mediated inhibition from the nodulus on the vestibular nuclei. This was the first example of a medication that could completely stop a persistent pathological nystagmus! This successful outcome arising from a fortuitous interaction at a scientific meeting emphasizes the importance of “broadening your horizons” by interacting with colleagues from afar. This case also illustrates the power of the control systems approach to clinical problems and, in this day of high technology, the importance of imaginative thinking with the help of just a paper and a pencil, especially when they are in the hands of someone like David Robinson.

There are many other examples of how studying the cerebellum and cerebellar patients has revealed much about how the brain works, and how we can better diagnose and treat patients with cerebellar diseases. The first descriptions of an unstable neural integrator came from studies of animals with experimental lesions in the flocculus, and in a patient with a paraneoplastic cerebellar degeneration [8, 22]. Recent studies in patients with acute strokes who had lesions isolated to the flocculus or to the paraflocculus (tonsil) have allowed us to pinpoint a role for these particular structures in the fine grain control of eye movements and the VOR [23–25]. These studies have led my close colleagues, David Newman-Toker, Jorge Katah and Ji-Soo Kim, and their collaborators, to develop better and critically needed algorithms for diagnosing patients with strokes in the brainstem and cerebellum [23–26]. Quantification of the VOR can be an important biomarker of progression of some forms of cerebellar disease and potentially a marker of response to treatment [27, 28]. Correlations of ocular motor behavior with findings on functional and structural imaging of the cerebellum have been a boon to our knowledge of the behaviors in which the cerebellum is involved [29]. Studies of patients with a curious neurological disorder (ocular-palatal tremor syndrome) associated with hypertrophy and degeneration of the inferior olive, have given insights into what happens when the cerebellum attempts to compensate for motor dysfunction with feedback about motor performance that is inaccurate. With the cerebellum playing a central role in the adaptive responses of the brain to disease and trauma, a knowledge of how the cerebellum promotes compensation for lesions elsewhere in the brain becomes a key pillar for developing better physical therapy programs for rehabilitation of brain-damaged patients [30–32].

With an almost daily increase in the knowledge of the genetics of cerebellar disease, ocular motor function is often the cornerstone of phenotypic classification and differential diagnosis (e.g., [33]). Particularly satisfying has been the identification of the genetic defect in two groups of patients that we studied in the 1970s. First, a large pedigree of patients with a late-onset, isolated cerebellar degeneration eventually turned out to have spinocerebellar ataxia type 6 (SCA6) with an abnormality in the calcium channel on chromosome 21 [16, 34]. I have followed four generations in one family with this syndrome. Secondly, the patients with slow saccades who were the basis of our local feedback model of control of saccades, turned out to have spinocerebellar ataxia type 2 (SCA2) with an abnormality on chromosome 12 (ATXN2 gene). In the last decade, my interests in the cerebellum led me to be a co-founding member of the multidisciplinary Johns Hopkins ataxia clinic generously supported by the Macklin Foundation. Patients come for a complete evaluation and management of their ataxia; a neurologist, geneticist, physical and occupational therapists, social worker, etc., all see the patient in the clinic on the same day to deliver expert, comprehensive and efficient clinical care.

### Collaborate!

**Fig. 3:**
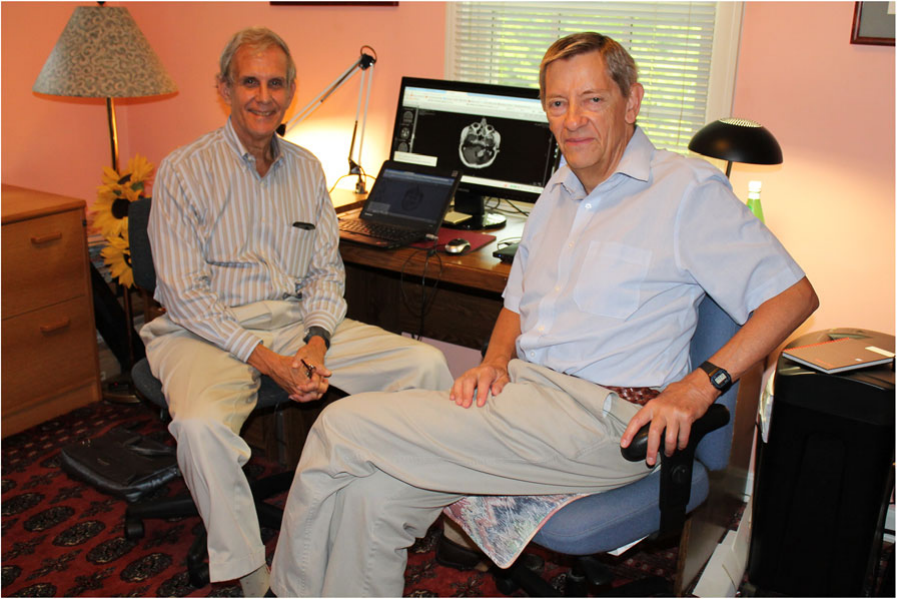
John Leigh (right) and Dave Zee working on the 5th edition of The Neurology of Eye Movements in Cleveland in 2014

**Fig. 4:**
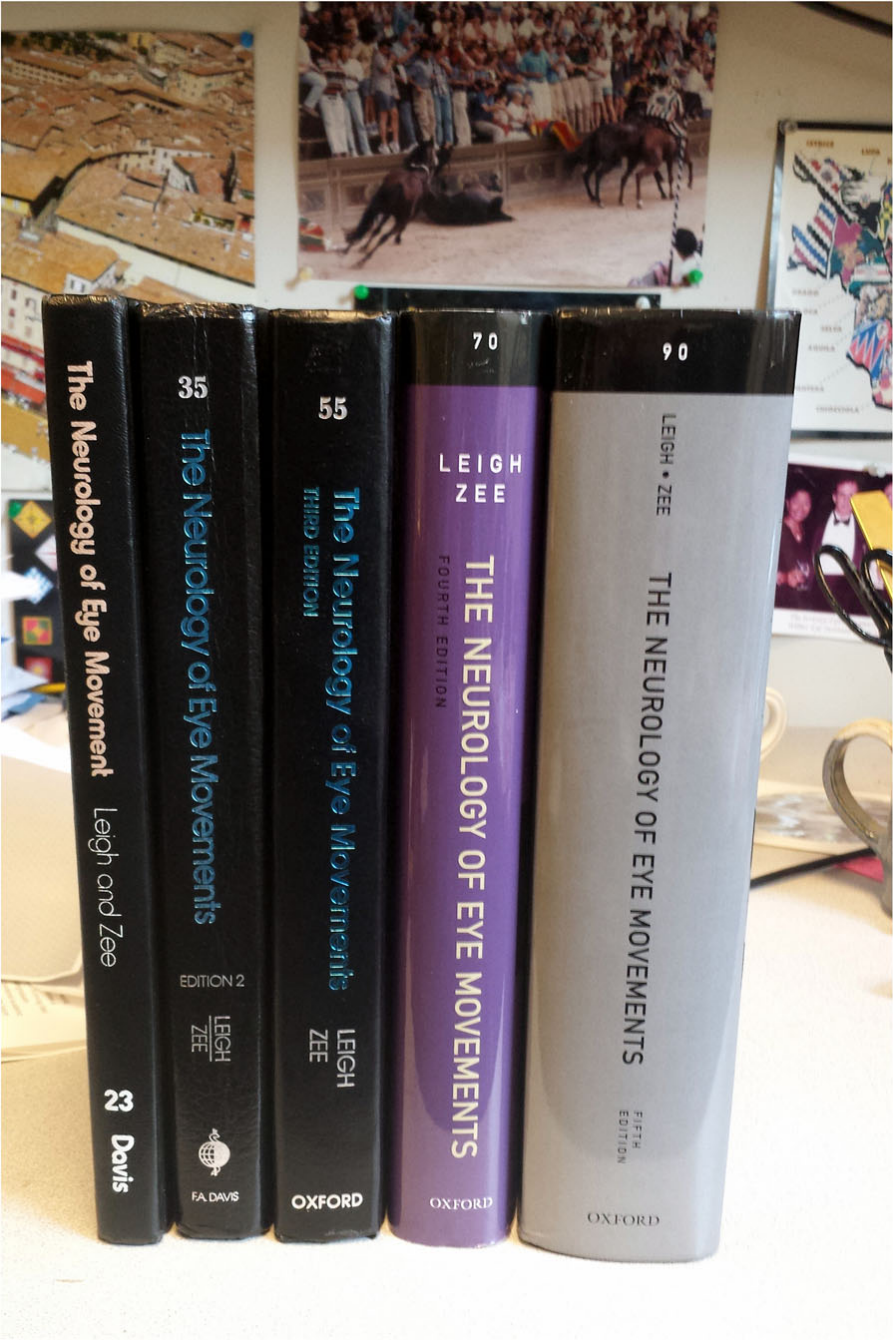
Five editions of Leigh and Zee, The Neurology of Eye Movements, the first in 1983 (left), the most recent in 2015 (right)

“Interact and collaborate with colleagues and trainees”. My close colleague, John Leigh, with whom I began working in the 1970s when he came to Hopkins as a postdoctoral fellow, said in the early 1980s that it was time to write a new book about eye movements. Dr. David Cogan’s “The Neurology of the Ocular Muscles” had its last edition published in 1966, and much new information and many novel approaches had emerged since then. So, after a little prodding, I agreed and the first edition of Leigh and Zee, “The Neurology of Eye Movements” appeared in 1983, and the most recent, fifth edition in 2015 (Figs. 3 and 4). “Write papers, or even a book to educate your colleagues.” The field has grown as has our book from 281 pages in the first edition to 1109 in the last, and more than 10,000 “selected” citations in the most recent edition! Videos and digital platforms for mobile devices have enhanced the use of this book, but it is remarkable that the fundamental concepts, largely derived from our early collaborations with Dave Robinson, survive relatively unchanged.

Many of my postdoctoral fellows and colleagues have gently but firmly pointed or rather pushed me in many different ways. Examples are ophthalmologists to strabismus, otolaryngologists to diseases of the inner ear, physical rehabilitation specialists to motor learning, adaptation and compensation, movement disorder neurologists to dystonia and tremor, bioengineers to models of nystagmus and other oscillations, and brilliant students who just brought their native intelligence and curiosity to the lab and clinic and raised important and often discomforting questions. Collaboration, free exchange of information, and coming out of your silo to see what else is around is at the core of scientific progress (e.g., [21]). Three individual sabbatical years at the National Eye Institute in Bethesda, working with Lance Optican, Ed Fitzgibbon, Christian Quaia and other colleagues in the laboratory of sensorimotor research (LSR), led to fruitful publications and changes in my research priorities [35–42]. Several different summers in which I spent a month at the University of Zurich in the laboratory of Dominik Straumann to revitalize my thinking, were vital to me for receiving a continuous RO1 individual research grant for 36 years. “Take sabbaticals” and “Persevere but be willing to change course when you should change course”.

**Fig. 5:**
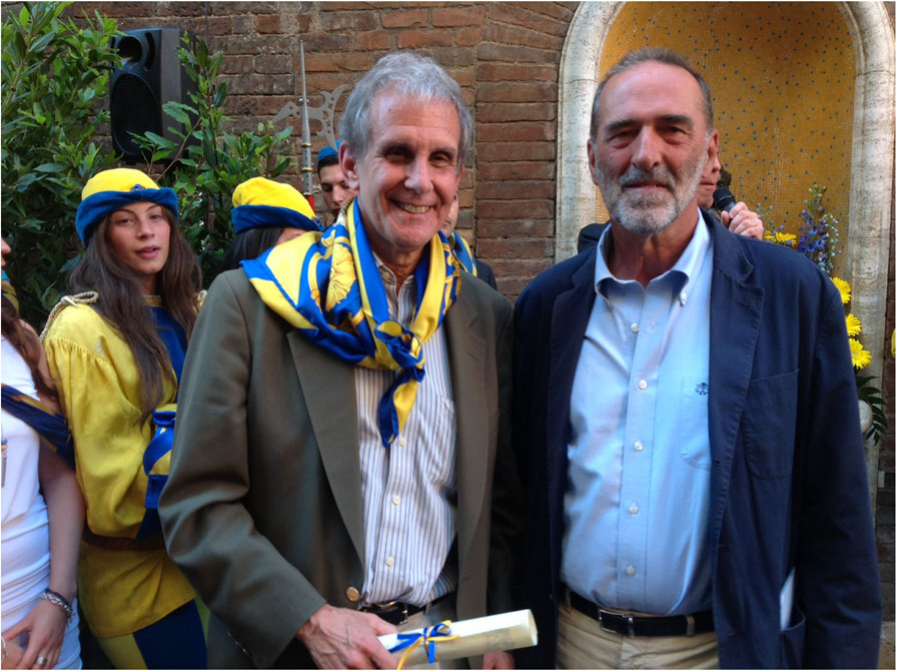
Long-term collaborators. David Zee and Daniele Nuti (right), Professor of Otolaryngology at the University of Siena in Italy have been meeting in Siena annually for more than 25 years. Dave joined Tartuca, Daniele’s contrada. There are 17 contradas (local districts) in Siena. They compete fierecely twice each year during the summer in a famous horse race (the Palio) three times around the town square. Here David Zee has just been “baptized” into the Contrada with about 25 others who were mostly under the age of 2 years

My own current research focus is on how magnetic fields stimulate the labyrinth and produce nystagmus, and what this tells us about how the brain adapts to vestibular disorders [43, 44]. This new area of research for me grew from a casual conversation with an Italian neuro-otologist, Vincenzo Marcelli, on one of many visits to the University of Siena where I have had a long-standing collaboration with Professor Daniele Nuti (Fig. 5). “Broaden your horizons. Meet colleagues and students from other countries and cultures”. One of the most rewarding aspects of my career has been collaborations, teaching and visits with close colleagues in countries all over the world. I have also been fortunate to collaborate for years with wonderful scientists and clinicians at Johns Hopkins: in biomedical engineering, ophthalmology, otolaryngology, neurology and neuroscience. Collegiality is a core tenet of the Hopkins experience.

### Why teach?

“Learn how to teach effectively and how to write concisely. Get feedback from mentors and students”. Working with students and trainees is also a pillar of scientific progress and personal satisfaction. As Sir William Osler emphasized, conveying new knowledge and stimulating people to learn about or jump into one’s field is perhaps the most fundamental and gratifying contribution that most of us can make in our academic lives. Students have forced me to learn about something new or to do something differently, or opened up a new way of thinking about a problem. You know you have succeeded as a teacher when you are learning more from your trainees than they from you. Teaching prods us to examine our frequently superficial understanding of key issues and concepts. When things are murky, teaching pushes us back to the drawing board. Every time we teach, we learn is not a hackneyed phrase but a genuine acknowledgement of a mainstay of academic life. Teaching allows us to disseminate knowledge to many scientists or physicians at once, and in the case of clinical audiences to affect the medical care immediately of hundreds or even thousands of patients. Perhaps one of the most important recent applications of our studies of the effects of cerebellar lesions on eye movements has been the development of algorithms to distinguish stroke in the cerebellum or brainstem from benign afflictions of the peripheral labyrinth in acutely vertiginous patients [26]. Teaching and stimulating students allows us to bring new blood into one’s field by attracting the smartest and most imaginative to follow our example. Teaching allows us to meet students and colleagues from all over the world, from different cultures with different approaches to medicine, to science and to life.

Learning to write concisely, too, is a core skill of an effective teacher. “Get feedback!” Dave Robinson’s notorious red pen with which he butchered my first drafts of a paper, and the stinging critiques from (usually) thoughtful reviewers (“if the reviewer did not understand what you wrote, it was your problem, not the reviewers”) have been painful but essential experiences in learning how to disseminate scientific knowledge effectively.

## Conclusions

Some of these personal recommendations for choosing and developing one’s scientific path are summarized in Table 1. In many ways, these suggestions follow on those from Francis Bacon, which Gordon Holmes offered as the best advice one could give, “Desire to seek, patience to doubt, fondness to meditate, slowness to assert, readiness to reconsider, carefulness to dispose and set in order” [45, 46].

## Additional files

Additional file 1: Cerebellar related publications. (DOCX 35 kb)

Additional file 2: Curriculum vitae, David S Zee. (DOCX 126 kb)

## Abbreviations

ARVO: Association for Research in Vision and Ophthalmology
PAN: periodic alternating nystagmus
VOR: vestibulo-ocular reflex
