# Supplementary material for: A neurologist and ataxia: using eye movements to learn about the cerebellum
Source: Cerebellum Ataxias. 2018 Feb 7;5:2. doi: 10.1186/s40673-018-0081-2 (PMC5804057; doi:10.1186/s40673-018-0081-2)
Supplement: Supplementary file 1 — Cerebellar related publications. (DOCX 35 kb) [file 40673_2018_81_MOESM1_ESM.docx]

# DAVID SAMUEL ZEE Cerebellum related publications

1. Zee, D.S., Friendlich, A. and Robinson, D.A., The mechanism of downbeat nystagmus. Arch. Neurol. 30:227-237, 1974.
2. Zee, D.S., Cogan, D.G., Robinson, D.A. and Engel, W.K., Analysis of eye movements in members of a family with familial late-onset cerebellar ataxia. TTTTTTransactions of the American Neurological Association, 100:98-103, 1975.
3. Zee, D.S., Optican, L., Cook, J.D., Robinson, D.A. and Engel, W.K., Slow saccades in spinocerebellar degeneration. Arch. Neurol., 33:243-251, 1976.
4. Zee, D.S., Yee, R.D., Cogan, D.G., Robinson, D.A. and Engel, W.K., Ocular motor abnormalities in hereditary cerebellar ataxia. Brain, 99:207-234, 1976.
5. Zee, D.S. and Robinson, D.A., A hypothetical explanation of saccadic oscillations. Ann. Neurol. 5:405-414, 1979.
6. Yamazaki, A. and Zee, D.S., Rebound nystagmus: an electro-oculographic analysis of a case with a floccular tumor. Brit J Ophthalmol., 63:782-786, 1979.
7. Zee, D.S. and Robinson, D.A., Clinical applications of oculomotor models. In: Topics in Neuro-Ophthalmology, Ed. H.S. Thompson, Williams & Wilkens, Baltimore, pp. 266-285. 1979.
8. Zee, D.S., Leigh, R.J. and Mathieu-Millaire, F., Cerebellar control of ocular gaze stability. Ann. Neurol., 7:37-40, 1980.
9. Leigh, R.J. and Zee, D.S., Eye movements of the blind. Invest. Ophthalmol. 19:327-331, 1980.
10. Halmagyi, G., Rudge, P., Gresty, M., Leigh, R.J. and Zee, D.S., Treatment of periodic alternating nystagmus. Ann. Neurol., 8:609-611, 1980.
11. Leigh, R.J., Robinson, D.A. and Zee, D.S., A hypothetical explanation of periodic alternating nystagmus: instability in the optokinetic-vestibular system. Ann. N. Y. Acad. Sci ., 374:619-635, 1981.
12. Zee, D.S., Yamazaki, A., Butler, P.H. and Gücer, G., Effects of ablation of the flocculus and paraflocculus on eye movements in primate. J. Neurophysiol., 46:878-899, 1981.
13. Zee, D.S., Cerebellar control of eye movements. In: Nystagmus and Vertigo: Clinical Approaches to the Patient with Dizziness, Eds. V. Honrubia and M. Brazier, Academic Press, pp. 241-249, 1982.
14. Zee, D.S., Ocular motor control: the cerebellum. In: Neuro-Ophthalmology, Vol. II, Eds. S. Lessell and J.T.W. van Dalen, Excerpta Medica, Amsterdam, pp. 136-147, 1982.
15. Zee, D.S. Ocular motor abnormalities related to lesions in the vestibulocerebellum in primate. In: Functional Basis of Ocular Motility Disorders, Eds. G. Lennerstrand, D.S. Zee and E.L. Keller, Pergamon Press, Oxford, pp. 423-430, 1982.
16. Zee, D.S. and Optican, L.M., Mechanisms of ocular oscillations. In: Tremor, Eds. L. Findley and R. Capildeo, MacMillan Press, Ltd., London, 1984.
17. Zee, D.S., New concepts of cerebellar control of eye movements. Otolaryngology-Head and Neck Surgery, 92:59-62, 1984.
18. Zee, D.S., A hypothetical explanation of congenital nystagmus. Biological Cybernetics, 50:119-134, 1984.
19. Lisberger, S.G., Miles, F.A. and Zee, D.S., Signals used to computer errors in the monkey vestibulo-ocular reflex: possible role of the flocculus. J. Neurophysiol. 52:1140-1153, 1984.
20. Optican, L.M., Zee, D.S., and Miles, F.A., Floccular lesions abolish adaptive control of postsaccadic ocular drift in primates. Exp. Brain Res. 64:596-598, 1986.
21. Zee, D.S., Brain stem and cerebellar deficits in eye movement control. Trans. Ophthalmol. Soc. UK., 105:599, 1986.
22. Zee, D.S., Eye movement disorders in cerebellar disease. ENG Report, (ICS Medical), March issue, 1987.
23. Hain, T.C., Zee, D.S. and Maria, B., Modification of the dynamics of the vestibuloocular reflex by head tilt in patients with cerebellar lesions. In: The Vestibular System Neurophysiologic and Clinical Research eds. M.D. Graham and J.L. Kemink, Raven Press, New York, pp. 217-223, 1987.
24. Hain, T.C., Zee, D.S. and Maria, B., Tilt-suppression of the vestibulo-ocular reflex in patients with cerebellar lesions. Acta Otolaryngol. 105: 13-20, 1988.
25. Lewis, R.F. and Zee, D.S., Ocular motor disorders associated with cerebellar lesions: pathophysiology and topical localization. Rev. Neurol . (Paris), 149:665-677, 1993.
26. Deuschl, G., Toro, C., Valls-Solé, J., Zeffiro, T., Zee, D.S., Hallett, M., Symptomatic and essential palatal tremor. 1. Clinical, physiological, and MRI analysis. Brain,117:775-788, 1994.
27. FitzGibbon, E., Calvert, P., Zee, D.S., Dieterich, M., and Brandt, T., Torsional nystagmus during vertical smooth pursuit. J. of Neuroophthalmology, 16:79-90, 1996.
28. Zee, D.S., Considerations on the mechanisms of alternating skew deviation in patients with cerebellar lesions. J. Vestib. Research, 6:395-402, 1996.
29. Straumann, D, Zee, D.S., Solomon, D., Three-dimensional analysis of upward drift in cerebellar downbeat nystagmus, in "Three-dimensional Kinematics of Eye, Head and Limb Movement". (M. Fetter, T. Haslwanter, H. Misslisch, D. Tweed, eds.), Harwood Academic Publishers, Amsterdam, 243-250, 1997.
30. Gomez, C.M, Thompson, R.M., Gammack, J.T., Perlman, S.L., Dobyns, W.B., Truwit, C.L., Zee, D.S., Clark, H.B., and Anderson, J.H., Spinocerebellar ataxia type 6: Gaze-evoked and vertical nystagmus, Purkinje cell degeneration, and variable age of onset. Ann. Neurol. 42:933-950, 1997.
31. Takagi, M., Zee, D.S., and Tamargo, R., Effects of lesions of the oculomotor vermis on eye movements in primate: saccades, J Neurophysiol., 80:1911-1931, 1998.
32. Walker, M.F. and Zee, D.S. Hyperventilation and downbeat nystagmus in cerebellar patients. Neurology, 53:1576-1579, 1999.
33. Walker, M.F. and Zee, D.S. Directional abnormalities of vestibular and optokinetic responses in cerebellar disease. Ann. N.Y. Acad. Sci. 871:205-220, 1999.
34. Takagi, M., Zee, D.S., Tamargo, R., Effects of lesions of the oculomotor cerebellar vermis on eye movements in primate: Smooth pursuit. J. Neurophysiol., 83:2047-2062, 2000.
35. Straumann, D., Zee, D.S., Solomon, D., Three-dimensional kinematics of ocular drift in humans with cerebellar atrophy. J. Neurophysiol., 83:1125-1140, 2000.
36. Goldberg, M.D., Landa, R., Lasker, A., Cooper, L., and Zee, D.S., Evidence of normal cerebellar control of the vestibulo-ocular reflex (VOR) in children with high functioning autism. J. Autism Dev Disorders,30:519-524, 2000.
37. Fife, T.D., Tusa, R.J., Furman, J.M., Zee, D.S., Baloh, R.W., Hain, T., Goebel, J., Frohman, E., Demer, J., Eviatar, L., Assessment: Vestibular testing techniques in adults and children. Neurology,55:1431-1441, 2000.
38. Walker, M., Zee, D.S., Cerebellar control of gaze, in Neuroophthalmology at the Beginning of the Millenium. Ed. James Sharpe, Medimond Publishing, New Jersey, pp 71-82, 2000.
39. Zee, D.S., Walker, M.F., Ramat, S., The cerebellar contribution to eye movements based upon lesions: binocular, three-axis control and the translational vestibulo-ocular reflex. Ann NY Acad. Sci. 956:178-189, 2002.
40. Zee, D.S., and Leigh, R.J., Ocular motor control -- normal and abnormal. In: Diseases of the Nervous System, eds., Asbury, A., McKhann, G.M. and McDonald, W.I, Mcarthur, J., Cambridge University Press 634-657, 2002.
41. Walker, M.F. and Zee, D.S., Three-axis eye movement abnormalities with cerebellar lesions. Ann NY Acad Sci, 978:547, 2002.
42. Walker, M.F., Steffen, H., Zee, D.S., Three-axis approaches to ocular motor control: a role for the cerebellum, in "Levels of Perception", eds. L. Harris, M. Jenkin. Springer-Verlag, 399-413. 2002.
43. Takagi, M., Tamargo, R., Zee, D.S., Effects of lesions of the cerebellar oculomotor vermis on eye movements in primate: binocular control. in, Neural Control of Space Coding and Action Production eds. Prablanc, C., Pélisson, D., Rossetti, Y., Progress in Brain Research, 142:19-33, 2003.
44. Ying, SH, Choi, SI , Lee, M , Perlman, SL , Baloh, RW , Toga, AW, Zee DS, Relative atrophy of the flocculus and ocular motor dysfunction in SCA2 and SCA6, Ann NY Acad Sci. 1039, 430-435, 2005.
45. Lasker; A.G. Isotalo, E.H., Zee, D.S. Predictive saccades to a regularly alternating target in cerebellar patients. Ann NY Acad Sci. 1039, 544-547, 2005.
46. Walker, M., and Zee, D.S., Asymmetry of the pitch vestibulo-ocular reflex in patients with cerebellar disease. Ann NY Acad Sci. 1039: 349-358, 2005.
47. Walker, M., and Zee, D.S., Cerebellar disease alters the axis of the high-acceleration vestibulo-ocular reflex, J. Neurophysiol.,94: 3417-3529, 2005.
48. Ying, S., Choi, S., Perlman, S., Baloh, R., Zee, D.S, Toga, A., Pontine and cerebellar atrophy correlate with clinical disability in SCA2, Neurology,66:424-426, 2006.
49. Ying, S. and Zee, D.S., Phoria adaptation after sustained symmetrical convergence: influence of saccades, Exp Brain Res, 171: 297-305, 2006.
50. Walker, M., Tian, J., Zee, D.S., Kinematics of the rotational vestibulo-ocular reflex: role of the cerebellum and implications for neural control of Listing’s law, J. Neurophysiol., 98:295-302, 2007.
51. Walker, M.F., Tian, J., Shan, X., Tamargo, R.J., Ying, H., and Zee, D.S., Effects of cerebellar lesions in monkeys on gaze stability. in Advances in Understanding Mechanisms and Treatment of Infantile forms of Nystagmus, eds. Leigh, RJ and Devereaux, MW. Oxford University Press, pp 55-60, 2008
52. Walker, M., Tian, J., Shan, X., Tamargo, R., Ying, H., Zee, D.S., Lesions of the cerebellar nodulus and uvula impair downward pursuit, J Neurophysiology, 100,1813-1823, 2008.
53. Walker, M, Tian, J, Shan, X, Tamargo, R, Ying, H, Zee, DS, Lesions of the cerebellar nodulus and uvula in monkeys: effect on otolith-ocular reflexes. Progress Brain Res, 171: 167-172, 2008.
54. Liao, K, Hong, S, Zee, DS, Optican, LM and Leigh, RJ. [Impulsive head rotation resets oculopalatal tremor: examination of a model](http://www.sciencedirect.com/science?_ob=GatewayURL&_method=citationSearch&_urlVersion=4&_origin=SDVIALERTHTML&_version=1&_uoikey=B7CV6-4T7WBSW-18&md5=9d8fb91ebac5d192ec249ca63bb7ef7f), Progress Brain Res, 171, 227-234, 2008.
55. Hong, S, Leigh, RJ, Zee, DS and Optican, LM, [Inferior olive hypertrophy and cerebellar learning are both needed to explain ocular oscillations in oculopalatal tremor](http://www.sciencedirect.com/science?_ob=GatewayURL&_method=citationSearch&_urlVersion=4&_origin=SDVIALERTHTML&_version=1&_uoikey=B7CV6-4T7WBSW-17&md5=a14f3025614675747f3c1208599408f5), Progress Brain Res, 171, 219-226, 2008.
56. Zee, DS, and Walker, M, Cerebellum and oculomotor control, in Squire, LR (ed), Encyclopedia of Neuroscience, third edition, vol. 2, pp 729-736, Elsevier, Press, 2009.
57. Shaikh, AG, Marti, S, Tarnutzer, AA, Palla, A, Crawford, TO, Straumann, D, Taylor, AM, Zee, DS, Gaze fixation deficits and their implication in ataxia-telangiectasia, J Neurol Neurosurg Psychiatry 80:855-864, 2009.
58. Walker, M, Tian, J, Shan, X Tamargo, R, Ying, H, Zee, DS, Enhancement of the bias component of downbeat nystagmus after lesions of the nodulus and uvula. Ann NY Acad Sci, 1164: 482-485, 2009.
59. Ying, SH, Landman, BA, Chowdhury, S, Sinofsky, AH, Gambini, A, Mori, S, Zee, DS, Prince, JL, Orthogonal diffusion-weighted MRI measures distinguish region-specific degeneration in cerebellar ataxia subtypes, J Neurology, 256: 1939-42, 2009.
60. [Xu-Wilson](Javascript:%20view_member('Haiyin',%20'Chen');), M, [Chen-Harris](Javascript:%20view_member('Wilsaan',%20'Joiner');), H, [Zee,](Javascript:%20view_member('David',%20'Zee');) DS, Shadmehr, R, Cerebellar contributions to adaptive control of saccades in humans, J Neuroscience, 29: 12930-39, 2009.
61. Shaikh AG, Hong S, Liao K, Tian J, Solomon D, Zee DS, Leigh RJ, Optican LM, Oculopalatal tremor explained by model with inferior olivary hypertrophy and cerebellar plasticity, Brain, 133: 923-940, 2010.
62. Du, A, Bennett, L. Zee, DS, Mori, S, Prince, JL, Ying, SH, Diffusion tensor imaging reveals disease-specific deep cerebellar nucleus changes in cerebellar degeneration, J Neurology, 257:1406-08, 2010.
63. Walker, M, Tian, J, Shan, X, Tamargo, R, Ying, H, Zee, DS, The cerebellar nodulus/uvula integrates otolith signals for the translational vestibulo-ocular reflex, PLoS ONE 5(11): e13981. doi:10.1371/ 2011.
64. Shaikh, AG, Marti, S., Tartnutzer, A., Palla, A., Crawford, TO, Straumann, D., Carey, JP, Nguyen, KD, Zee, DS, Ataxia-telangiectasia: a “disease model” to understand cerebellar control of vestibular reflexes, J Neurophysiology, 105: 3034-41, 2011.
65. Wan, J, Mamsa, H, Johnston, JL, Spriggs, EL, Singer, HS, Zee, DS, Al-Bayati, AR, Baloh, RW, Jen, JC, Large Genomic Deletions in CACNA1A Cause Episodic Ataxia Type 2, Front.Neur. 2:51. doi: 10.3389/fneur.2011.00051, 2011.
66. Kheradmand, A, Zee, DS, Cerebellum and Ocular Motor Control, Front.Neur. 2:53. doi: 10.3389/fneur.2011.00053, 2011.
67. Lopez, J, Zee DS, Levy, L, Eye closure and oculopalatal tremor, Neurology, 77:1929, 2011.
68. Jung, BC _,_ Choi, SI, Du, AX, Cuzzocreo, JL, Ying, HS, Landman, BA, Perlman, SL, Baloh, RW, Zee, DS, Toga, AW, Prince, JL, Ying, SH, MRI shows a region-specific pattern of atrophy in spinocerebellar ataxia type 2. Cerebellum, 11: 272-279, 2012.
69. Shaikh, A, Palla, A, Marti, S, Olasagasti, I, Optican, LM, Zee, DS, Straumann, D, Role of cerebellum in motion perception and vestibulo-ocular reflex – similarities and disparities, The Cerebellum,12:97-107, 2013.
70. Park, H-K, Kim, J-S, Strupp, M, Zee, DS, Isolated floccular infarction: impaired vestibular responses to horizontal head impulse, J Neurology, 260:1576-82, 2013.
71. Shaikh, A, Zee, DS, Mandir, AS, Lederman, HM, and Crawford, TC, Disorders of upper limb movements in ataxia-telangiectasia, PLoS ONE, 8(6), e67042 2013.
72. Shaikh, A, Marti, S, Tarnutzer, AA, Crawford, TO, Zee, DS, Straumann, D, Effects of 4-aminopyridine on nystagmus and vestibulo-ocular reflex in ataxia-telangiectasia, J Neurology, 260:2728 – 2735, 2013
73. Lee, S-H, Park, S-O, Kim, J-S, Kim, H-J, Yunusov, F, Zee, DS, Isolated unilateral infarction of the cerebellar tonsil: ocular motor findings. Ann Neurol 75:429-34, 2014.
74. Kim, HG, Kim, JS, Choi, JH, Choi, KD, Zee DS, Rebound upbeat nystagmus after lateral gaze in episodic ataxia The Cerebellum, published online January 14, 2014.
75. Huh, YE, Kim, J-S, Kim, H-J, Park, S-H, Jeon, BS, Kim, J-M, Chu, JW, Zee DS. Vestibular performance during high-acceleration stimuli correlates with clinical decline in SCA6, Cerebellum, 14:284-9, 2015.
76. Patel, VR, Zee, DS, The cerebellum in eye movement control: nystagmus, coordinate frames and disconjugacy, Eye, 29:191-195, 2015.
77. Umeh, C., Polydefikis, M., Chaudhry, V. and Zee, DS, Sweat gland denervation in cerebellar ataxia with neuropathy and vestibular areflexia syndrome, Movement Disorders, Clinical Practice, 4: 46-48, 2016.
78. Khehadmand, A, Kim, J-S, Zee, D, Cerebellum and Oculomotor Deficits, in Essentials of Cerebellum and Cerebellar Disorders, editors Gruol, D et al., Springer, pp. 471-6, 2016.
79. Lee, SU, Choi, JY, Kim, HJ, Park JJ, Zee, DS, Kim, JS, [Impaired tilt suppression of post-rotatory nystagmus and cross-coupled head-shaking nystagmus in cerebellar lesions: image mapping study](http://www.ncbi.nlm.nih.gov/pubmed/26969184), The Cerebellum, 16:95-102, 2017.
80. Shaikh, AG, Wong, AL, Optican, LM, and Zee, DS, Impaired motor learning in a disorder of the inferior olive: Is the cerebellum confused? The Cerebellum, 16:158-167, 2017.
81. Schiess, N, Zee, DS, Siddiqui, KA, Szolics M, El Hattab, AW, Novel PNKP Mutation in siblings with Ataxia-Oculomotor Apraxia Type 4, J. Neurogenetics, 31: 23-25, 2017.
82. Yacovino, DA, Akly, MP, Leonel, L, Zee, DS, The floccular syndrome: dynamic changes in eye movements and vestibulo-ocular reflex in isolated infarction of the cerebellar flocculus, The Cerebellum, published on line, 2017
83. Kronenbuerger, M, Olivi, A, **Zee**, DS, A picture is worth a thousand words: Positional vertigo and vertical nystagmus in medulloblastoma, Neurology, in press, 2017.
84. Oh, EH, Lee , J-H, Shin, J-H, Kim, H-S, Kim, J-S, Kim, H-J, Choi, S-Y, Choi, K-D, **Zee**, DS, Cho, J-H, Patterns and modulations of Pendular nystagmus in a family with hereditary spastic paraplegia, J Neurological Sci. 383: 169-173, 2017.
85. Tehrani, AS, Kattah, J, Kerber, K, Gold, D, Zee DS, Urrutia, V, Newman-Toker, D, Diagnosing Stroke in Acute Dizziness and Vertigo: Pitfalls and Pearls, Stroke, in press, 2017
86. Choi, J-Y, Glasauer, S, Choi, SY, Kim, JH, Zee, DS, Kim, J-S, Apogeotropic central positional nystagmus: Characteristics and mechanism, Brain, in press, 2017.
