# Supplementary material for: A neurologist and ataxia: using eye movements to learn about the cerebellum
Source: Cerebellum Ataxias. 2018 Feb 7;5:2. doi: 10.1186/s40673-018-0081-2 (PMC5804057; doi:10.1186/s40673-018-0081-2)
Supplement: Supplementary file 2 — Curriculum vitae, David S Zee. (DOCX 126 kb) [file 40673_2018_81_MOESM2_ESM.docx]

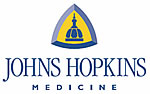


# CURRICULUM VITA

# DAVID SAMUEL ZEE January 2018

**DEMOGRAPHICS**

Office Address

Department of Neurology

Pathology 2-210

The Johns Hopkins Hospital

600 N. Wolfe St.

Baltimore, Maryland 21287-6921

(443) 287-4625 (voice)

(410) 614-1746 (fax)

Email: [davidsamuelzee@gmail.com](mailto:davidsamuelzee@gmail.com)

[Web page](http://vorlab.jhu.edu/)

**EDUCATION**:

1961-65 Bachelor of Arts (Chemistry), Northwestern University, Evanston, Illinois

1965-69 Doctor of Medicine, The Johns Hopkins University, Baltimore, Maryland

**POSTGRADUATE TRAINING**:

1969-70 Intern (Medicine), New York Hospital, Cornell University

1970-73 Resident (Neurology), The Johns Hopkins Hospital

**PROFESSIONAL EXPERIENCE:**

1973-75 Commissioned Officer, U.S. Public Health Service, NINDS, N.I.H.

1975-78 Assistant Professor of Neurology and Ophthalmology

The Johns Hopkins University

1978-85 Associate Professor of Neurology and Ophthalmology

The Johns Hopkins University

1985- Professor of Neurology (Primary Appointment, Endowed), Ophthalmology, Otolaryngology and Head and Neck Surgery, and Neuroscience, The Johns Hopkins University, School of Medicine

1982, 1991, 2005 Sabbatical Leaves, National Eye Institute, N.I.H.

**HONORS AND AWARDS:**

Phi Beta Kappa, Northwestern University, 1965

Henry Strong Denison Scholar, The Johns Hopkins University, 1967-68

Teacher-Investigator Award, National Institute of Neurological Diseases and Stroke, 1975-1980

*Inaugural* Frank R. Ford Award for Outstanding Clinical Teaching of Neurology, 1979

Research Career Development Award, National Eye Institute, 1980-1985

*Inaugural* Visiting Professor of Neurological Education, Mayo Clinic, Rochester, Minnesota, 1980

Merit (10-year research) RO1 Award, National Eye Institute, N.I.H., 1987-1997

Alpha Omega Alpha, Johns Hopkins Alumnus, 1991

Visual Sciences B, N.I.H. Study Section, 1989-93, Chair, 1992-93

Research to Prevent Blindness Manpower Award, 1993-1995

Hallpike-Nylén Medal of the Bárány Society, Uppsala, Sweden, 1994

Ottorino Rossi Award and Medal of the University of Pavia, Pavia, Italy, 1994

Frank R. Ford Award for Outstanding Clinical Teaching of Neurology, 1994

Member, National Advisory Eye Council, N.I.H, 1996-1999

National Health Information Awards (Honoring the Nation's Best Consumer Health Information, Programs and Materials), third place, for educational videotapes on dizziness (2000)

The Johns Hopkins School of Medicine Professor's Award for Distinction in Teaching in the Clinical Sciences (2001-2002)

*Inaugural* H. Houston Merritt Award and Lecture, American Academy of Neurology, April, 2003

Counselor, American Neurological Association, 2003-06

Bielschowsky prize (German Strabismus Society) to a coauthor for two papers in Investigative Ophthalmology Visual Science (Steffen et al, 2000, 2002)

Clinical and basic ocular motor research: an international meeting in Siena, Italy, July, 2004, in my honor

‘Master clinician award’, Johns Hopkins senior neurology residents, June, 2005, 2008, 2011

Department of Medicine Osler House staff award in recognition of outstanding teaching contributions, 2011

Koetser Foundation for Brain Research Prize, University of Zurich, Sept 2013

Neurology student clerkship outstanding lecturer award, 2013

Annual luncheon for teachers of neurology to medical students named “The David Zee Neurology Teaching Awards Ceremony” 2017.

VEDA (Vestibular Disorders Association) “Champion of Vestibular Medicine Award”, 2017.

Endowed Professor of Neurology, Johns Hopkins University, 2017.

**NAMED LECTURES** (30 including 4 inaugural lectures)

J. L. Silverside Lecture, University of Toronto, Toronto, 1986.

Morris Bender Lecture, Mt. Sinai Medical School, New York, 1992

Jerome Merlis Lecture, University of Maryland, Baltimore, 1992

Prince Lecture and Visiting Fellow, Northwestern University, Chicago, 1993

J. Clifford Richardson Lecture, Canadian Neurosciences Congress, Toronto, 1993

AAN Lecture at the Mexican Academy of Neurology, Oaxaca, Mexico, 1993

Ottorino Rossi Lecture, University of Pavia, Italy, 1994

*Inaugural* Swithin Meadows Lecture, National Hospital, Queen Square, London, 1995

Speaker of the Royal College of Physicians and Surgeons of Canada, Toronto, 1996

Norman Allen Lecture, Ohio State University Medical School, Columbus, 1997

*Inaugural* H. Houston Merritt Lecture, American Academy of Neurology, Honolulu, 2003

Professor G. Arjundas Oration, Madras Medical College, Chennai, India, 2005

Ramasamy Dayar Oration in Neuroscience, Ramachandra Medical College, Chennai, India, 2005

*Inaugural* Alfred Kestenbaum Lecture, University of Wurzburg, Germany, 2005

‘Brain’ lectures (National Hospital, Moorefield’s Eye Hospital, Charring Cross), London, 2006

Hartwell Thompson Lecture, University of Connecticut, Hartford, 2006

McNally Lecture, McGill University, Montreal, 2008

Dharmapuri Endowed Lecture, Sankara Nethralaya Eye Hospital, Chennai, India, 2009

Guy Williams Lecture, Cleveland Clinic, 2009

Pfizer Visiting Professor, University of Illinois, Peoria, 2010

Morris Bender Lecture, Mt. Sinai Medical School, New York, 2010

Lord Adrian Lecture, Cambridge University, England, 2012

Michael Sanders Lecture, Royal Society of Medicine, London, 2012

Irwin Levy Lecture, Washington University, St. Louis, 2012

Ed Marres Lecture, Maastricht University, The Netherlands, 2013

Koetser Foundation for Brain Research – Award lecture, Zurich, 2013

David Tomlinson Lecture, University of Toronto, 2014

*Inaugural* James Sharpe Memorial Lecture, University of Toronto, 2014

Kilian J. and Caroline F. Schmitt Lecture, University of Rochester, 2015

Loris and David Rich Lecture, University of Alabama, Birmingham, 2016

**ADDITIONAL MAJOR INVITED LECTURES:**

World Congress of Ophthalmology, Satellite Symposium, Tokyo, Japan 1978

Pediatric Neuroophthalmology Course, Sydney, Australia, 1981

Functional Basis of Ocular Motility Disorders, Stockholm, Sweden, 1981

Physiological and Pathological Aspects of Eye Movements, Belgium, 1982

Mechanisms of Tremor, Marbella, Spain, 1983

Boorhaven Teaching Courses, Leiden, Holland, 1984, 1985

American Neurological Association, Chicago, 1984

Trinational Conference on Motor Control, Tel Aviv, Israel, 1985

Adaptive Processes in Visual and Oculomotor Systems, Asilomar, California, 1985

Japanese Society for Equilibrium Research, Kobe, Japan, 1987

Australian Neuroophthalmological Society, Melbourne, Australia, 1987

Pan-Asian Oceanic Otolaryngological meeting, New Delhi, India, 1987

Colombian Neurological Society, Bogota, Colombia, 1988

French Neurological Society, Paris, France, 1988

European Conference on Eye Movements, Pavia, Italy, 1989

Belgian Neurological Society, Bruges, Belgium, 1990

Bárány Society, Tokyo and Sendai, Japan, 1990

Vestibular Pharmacology Conference, Florence, Italy, 1991

Bárány Society, Prague, Czechoslovakia, 1992

French Neurological Society, Paris, France, 1992

Brazilian Neurological Congress, Porto Alegre, Brazil, 1992

Japanese Society for Equilibrium Research, Maebashi, Japan, 1992

Charles University, Prague, Czech Republic, 1993

Pan Hellenic Neurology Congress, Thessalonica, Greece, 1993

Neurootology Symposium, Siena, Italy, 1993

International Neuroophthalmology Society, Freiburg, Germany, 1994

Neuroophthalmology Symposia, Buenos Aires and Rosario, Argentina, August, 1994

Mexican Academy of Neurology, Villahermosa, Mexico, October, 1994

British Ocular Motor Group, London, England, December, 1994

South African Neurological Association, Bloemfontein, South Africa, March, 1995

European Neuroophthalmology Society, Antwerp, May, 1995

Swedish Ophthalmology Society, Stockholm, May, 1995

Neurootology Workshop, Pamplona, Spain, September, 1995

Conference on Vestibular Cortex, Strasbourg, France, October, 1995

Japanese Neuroophthalmology Society, Tokyo, Japan, November, 1996

Hokkaido Neurology Society, Sapporo, Japan, November, 1996

Niigata Ophthalmology Society, Niigata, Japan, November, 1996

Neuroophthalmology and Neurootology symposium, Buenos Aires, Argentina, December, 1996

Neurootology symposium, World Congress of Neurology, Buenos Aires, Argentina September, 1997

Symposium on Noninvasive Monitoring in Intensive Care, Hamburg, Germany, January, 1998

Taiwanese Annual Neurology Meeting, Tainan, Taiwan, March, 1998

Neurootology – Neuroophthalmology Courses, Pamplona, Spain, March, 1998

Otolith Function in Spatial Orientation and Movement, Zurich, Switzerland, May, 1998

Neurogenetics Conference, Terni, Italy, July, 1998

Influence of Virtual Images on Human Factors, Tokyo, Japan, May, 1999

Vestibular Disorders Symposium, Firenze, Italy, June, 1999

Eye Movement Symposium, Beijing, China, July, 1999

Pan-American Congress of Neurology, Cartagena, Columbia, October, 1999

Iberoamericano Neurootology Congress, Rosario, Argentina, April, 2000

South African Specialist Course for Neurologists, Cape Town, South Africa, August, 2000

International Neuroophthalmology Society, Toronto, Canada, September, 2000

Eye Movement Symposium honoring Han Collewijn, Amsterdam, Holland, September, 2000

Visiting Professor, Guys Hospital – Johns Hopkins Exchange, London, October 1-28, 2000, with formal lectures at Guys, St. Thomas, Kings Cross, Moorefield's, Charring Cross, the National Hospital at Queens Square, and the Departments of Physiology and of Neurology at Oxford University

Eye Movement Symposium honoring Bernard Cohen, Seeon, Germany, March, 2001

Eye Movement Symposium honoring the inauguration of Inserm Unit 594 in Lyon, France, March, 2001

Disorders of Postural Control and Vestibular Function, Pamplona, Spain, May, 2001

Italian Congress of Otorhinolaryngology, Lecce, Italy, July, 2001

Philadelphia Neurological Society, January, 2002

International Neuroophthalmology Society, Buenos Aires, Argentina, May 2002

Swiss Neurological Society, Zug, Switzerland, May 2002

Japanese Neuroscience Society, Tokyo, Japan, July 2002

Erasmus University, Rotterdam, The Netherlands, Neuroscience Seminar Series, September, 2002

Bárány Society, Seattle, Washington, September, 2002

German Ophthalmological Society meeting on eye movements and vision, Heidelberg, Germany, October, 2002

German Society for Clinical Neurophysiology, Keynote lecture, Lübeck, Germany, October, 2002

Eye Movement Symposium honoring Ulrich Büttner, Wildbad-Kreuth, Germany, April, 2003

Agarini Foundation, Courses in Neuroscience, “Ataxias”, Alviano, Italy, May 2003

XVII Congreso Iberoamericano Otoneurologia, Madrid, Spain, June, 2003

University of Cadiz, teaching courses, Cadiz, Spain, June, 2003

American Headache Society, Chicago, Ill. June, 2003

100P^thP^ Meeting of the Chugoku-Shikoku Ophthalmology Society, Okayama, Japan, October, 2003

Department of Ophthalmology, Kawasaki University, Kurashiki, Japan, October, 2003

Strabismus Update, Symposium in honor of Gunner Lennerstrand, Stockholm, Sweden, January, 2004

Neuroophthalmology Symposium honoring Dr. Shirley Wray, Boston, Massachusetts, April, 2004

Club Latin American Neuroophthalmology, Mendoza, Argentina, May, 2004

‘Expert’ Neuroophthalmology Course, Zurich, Switzerland, July, 2004

Brazilian Neurology Congress, Brasilia, Brazil, October, 2004

Indian Neurological Society, Chennai, India, January, 2005

Department of Neurology, Bombay, India, January, 2005

International Neuropsychology Society, St. Louis, February, 2005

Korean Balance Society, Keynote lectures, Seoul, South Korea, June, 2005

Department of Psychology and Neuroscience program, Seoul National University, South Korea, June, 2005

Department of Neurology, Seoul National University Hospital, South Korea, June, 2005

World Congress of Otolaryngology, Rome, Italy, June, 2005

Association of Colombian Neurologists, Medellin, Colombia, August, 2005

Neuroscience Institute Congress, Lima, Peru, August, 2005

Bielschowsky Society, Lecture and honorary membership, Giessen, Germany, November, 2005

Symposium: Cortical control of higher motor cognition, Lübeck, Germany, May, 2006

European Society Neurology, Satellite symposium: Vestibular disorders, Lausanne, Switzerland, May, 2006

Symposium in honor of Pr. Dr. Johannes Dichgans, The Neurobiology of Eye Movements, Tübingen, Germany, July, 2006

International Neuroophthalmology Society, Tokyo, Japan, November, 2006

Riken Institute, Tokyo, Japan, November, 2006

Symposium in honor of Pr. Dr. Klaus Hess, Zurich, Switzerland, December, 2006

Clinical Neuroscience Update Teaching Course, Calcutta, India, Apr 2007

Mexican Academy of Otolaryngology, Chihuahua, Mexico, May 2007

Symposium on Neurootology, INEBA (Institute of Neurosciences), Buenos Aires, Argentina, May 2007

International Congress on Movement Disorders, Expert Course, Istanbul, Turkey, June, 2007

Israeli Neurootology Society, Haifa, Israel, October, 2007

International Teaching Course on Vestibular Disorders, Lisboa, Portugal, November, 2007

Symposium on eye movements in honor of Jean Buettner-Ennever, London, England, December, 2007

International Workshop on the Science of Balance Rehabilitation, Phoenix, co-organizer, January, 2008

Barany Society, Satellite symposium, honoring Professor Y. Shinoda, Ohtsu, Japan, April, 2008

Barany Society, Teaching course on vestibular disorders, Kyoto, Japan, May, 2008

XXI Congress Pan-American ORL society, Quito, Ecuador, June, 2008

Symposium honouring T. Brandt, Basic and Clinical Aspects of Vertigo and Dizziness, Seeon, Germany, June, 2008

Singapore Neuroscience Institute and Singapore National Eye Hospital, visiting lectures, September, 2008

Neuroophthalmology Society of Australia, Keynote lecture Canberra. September, 2008

German Ophthalmology Society (DOG), Berlin, Germany, September, 2008

Recherche en Oculo-Motricité, Nantes, France, September, 2008

Symposium on Eye Movements in honor of Albert Fuchs, Ashland, Oregon, October 2008

International Neurootology course and meeting, Mexico City, Mexico, October, 2008

Laboratory of Neurophysiology, UCL, Brussels, Belgium January, 2009 (seminar and PhD thesis jury)

Neuroophthalmology Update Course, Nethralaya Eye Hospital, Chennai, India, January, 2009

Symposium on Eye Movements in honor of Fred Miles, Oxford, England, April, 2009

Italian Society for Otorhinolaryngology, Rimini, Italy, May, 2009

International Congress on Movement Disorders, Teaching courses, Paris, France, June, 2009

Masters Course, Neurootology, University of Siena, Italy, July, 2009

Forum for Indian Neurological Education: teaching courses on neuroophthalmology and neurootology, Mumbai, India, September, 2009

Neuroophthalmology teaching course in honor of James Sharpe, Toronto, Canada, 2009

World Congress of Neurology (WCN), Bangkok, Thailand, October, 2009

National Congress of Turkish Neurologists, Antalya, Turkey, November, 2009

Plenary lecture, Neuroscience symposium celebrating 40 year anniversary of National Eye Institute, Bethesda, November, 2009

Universite Paris 6, Symposium on eye movements and PhD jury, Paris, France, December, 2009

Mexican Audiological Medicine Congress, Puebla, Mexico, March, 2010

Satellite symposium to Neural Control of Movement meeting honoring Ed Keller, Naples Florida, April, 2010

International Neurotology Symposium, Keynote Lecture, Matera, Italy, May, 2010

Royal Academy of Ophthalmology, Liverpool, England, May, 2010

Department of Neurology, Newcastle University, England, May, 2010

International Neuroophthalmology Society (INOS), Invited lecture and session chair, Lyon, France, June, 2010

Neuro-Ophthalmology Teaching Course, Invited lecture, Siena, Italy, June, 2010

International Strabismus Symposium, Invited lecture and closing lecture Stockholm, Sweden, June, 2010.

Barany Society, Teaching course on vestibular disorders, Reykjavik, Iceland, August, 2010.

University of Siena, Special lectures in Neuroscience for graduate students, Siena, Italy, October, 2010.

Forum for Indian Neurological Education: teaching courses on neuroophthalmology and neurootology, Chennai, India, November, 2010.

“NeuroPunta” Uruguay neurology congress, keynote lecture and workshop participant, Punta del Este, Uruguay, December, 2010

Institut du Cerveau et de la Moelle Epiniere (ICM), Invited lecture, Paris, France, March, 2011

International Symposium: Basic and Clinical Ocular Motor and Vestibular Research, honoring R. John Leigh, Teaching course, Buenos Aires, Argentina, March, 2011.

Biannual National Congress of Neurology, Keynote lecture, Borovets, Bulgaria, May, 2011

International Center of Biocybernetics, Saccadometry, Warsaw, Poland, June, 2011

International Congress on Movement Disorders, Teaching course, Toronto, Canada, June, 2011

Neuroophthalmology and Multiple Sclerosis, Teaching course, Siena, Italy, June, 2011

III International Conference on Balance Disturbances, Teaching course, Warsaw, Poland, September, 2011

Society of Italian Neurologists, Teaching course, Turin, Italy, October, 2011

Hong Kong Neurology Society, keynote speaker, Hong Kong, October, 2011

Forum for Indian Neurological Education: teaching courses on neuroophthalmology and neurotology, Calcutta, India, November, 2011

World Congress of Neurology (WCN), keynote lecture, teaching course, and scientific presentation, Marrakech, Morocco, November, 2011

Oxford University, Neurology Grand Rounds, Radcliffe Hospital, January, 2012

Royal College of Medicine, Neuroscience lecture, London, January, 2012

University of Ottawa, Visiting Professor, Canada, March, 2012

Dutch Ophthalmological Society, Groningen, Keynote lecture, The Netherlands, March, 2012

WHO conference on core values in vestibular disturbances, Munich, Germany, May, 2012

University of Siena, Teaching course, Eye movements and nystagmus, Siena, Italy, May, 2012

International Neuroophthalmology Society (INOS), Invited lecture and session chair, Singapore, June, 2012

Forum for Indian Neurological Education: teaching courses on neuroophthalmology and neurootology, Invited speaker, Agra, India, August, 2012

Society for French Neurology, Invited lecture, Paris, October 2012

Mediterranean Society of Otology and Audiology, Keynote lecture, Naples, Italy, October, 2012

Vertigo Academy International, Keynote lecture, Antalya, Turkey, November, 2012

Visiting Professor, Department of Ophthalmology, Boston Children’s Hospital, Harvard, Boston, 2012

Masters course in movement disorders, Groningen University, The Netherlands, December, 2012

Masters course in vestibular disorders, Maastricht University, The Netherlands, January, 2013

Invited speaker and congress chairman, The vestibular system, honoring Daniele Nuti, Siena, Italy, April, 2013

Invited speaker, Neurotology 2013, Ospedale San Raffaele, Milan, Italy, April, 2013

Invited speaker, Workshop on eye movements, Institut du Cerveau et de la Moelle Epiniere (ICM), Paris, June, 2013

Neuro-Ophthalmology Teaching Course, Invited lecture, Siena, Italy, June, 2013

Invited speaker, Symposium on Interactions in Vision, honoring Hugh Wilson and Marty Steinbach, York University, Toronto, June, 2013

Keynote lecture, NIH (NINDS) summer student symposium, July, 2013.

Keynote lecture, Neural Control of Movement Society, Amsterdam, The Netherlands, April, 2014

Plenary lecture, Barany Society, Buenos Aires, Argentina, May, 2014

Invited speaker, Vestibular rehabilitation symposium, Tigre, Argentina, May, 2014

Invited speaker, Swiss Society of Clinical Neurophysiology, Zurich, June, 2014

Invited speaker, Practical Neuro-ophthalmology, Update meeting of European Neuro-ophthalmology Society (E NOS), Zurich, June, 2014.

Masters course in vestibular neurology, co-teacher, Mexico City, Mexico, August, 2014

Invited speaker, Cambridge Ophthalmology Symposium, Cambridge, England, September 2014

Invited speaker, Forum for Indian Neurological Education: teaching courses on neuroophthalmology and neurootology, Pune, India, September, 2014

Masters course in vestibular disorders, University of Bern, Switzerland, February, 2015.

Keynote lecture, PanAfrican Federation of Otolaryngological Societies (PAFOS), Luxor, Egypt, March 2015

Workshop in vestibular disorders, University of Cairo, Egypt, March, 2015

ENT summit in vestibular disorders, Marrakech, Morocco, April, 2015

Keynote speaker, Vertigo Academy International II, Moscow, May, 2015

Invited speaker, Health effects from MRI-related magnetic fields, Utrecht, The Netherlands, June, 2015

Invited speaker, Five-day course in vestibular disorders, University of the Andes, Santiago, Chile, Sept, 2015

Keynote lecture, Japanese Neuro-Ophthalmology Society, Tokyo, Nov, 2015,

Invited lectures, Necker Children’s Hospital and Quinze-Vingts Eye Hospital, Paris, March 2016

Plenary Lecture, Barany Society Meeting, Seoul, Korea, June 2016,

Plenary lecture, Congresso Nazionale del Cenacolo Italiano Di Audiovestibologia, Chieti, Italia, June 2016

Invited speaker, 41^st^ Congresso, Conventus Societas, ORL Latina, Turino, Italy, July 2016

Keynote lecture, SSBP (Society for study of behavior phenotypes) conference, Siena, Italy, Sep 2016

Invited speaker, Cultural Week, Vestibular “state of the art”, Agropoli, Italy, Sept 2016

Invited speaker, Festschrift for Chris Kennard, Oxford University, England, Sept 2016

Invited speaker, Symposium: The vestibular system: sensory, motor and cognitive functions, Munich, Oct 2016

Keynote lecture, 5^th^ Belgrade Balance Forum, Belgrade, Serbia, April 2017

Invited speaker, 5^th^ International Vestibular Disorder Course and Workshop, Granada, Spain, June 2017

Invited speaker, World Congress, International Federation of Otolaryngology Societies, Paris, June 2017

Gordon Conference on Eye Movements, plenary lecture introducer, Lewiston, Maine, July 2017

Invited speaker, Forum for Indian Neurological Education: teaching courses on neuroophthalmology and neurootology, Mumbai, India, August, 2017

Invited speaker, “Teorico-Pratico Di Audiologica e Vestibologia”, Benevento, Italy, September 2017

Invited speaker, Korean Neurological Association, Seoul, November 2017

Invited speaker, Chonnam National University, Gwangju, Korea, November 2017

Invited speaker, 11^th^ GCC and 8^th^ Emirates Otolaryngology Conference, January 2018.

**RESEARCH INTERESTS:**

Normal and abnormal ocular motor, vestibular and cerebellar physiology

**ACTIVE GRANTS:**

Leon Levy Foundation, Studies of vestibular perception (support of junior faculty member) 2012-2018

N.I.H AVERT Acute Video-oculography for Vertigo in Emergency Rooms for Rapid Triage

9/16/14 - 8/31/19 U01-DC01378 Co-investigator

**RECENTLY COMPLETED GRANTS:**

N.I.H. Oculomotor Disorders: Clinical and Experimental Studies (1976-2012) R01-EY01849, Principal Investigator

N.I.H. MRI induced Vertigo (2011-2013) R21-DC11919, Principal Investigator

N.I.H. Ocular Motor Adaptation in Health and Disease (2008-2014) RO1-EY019347, Co-investigator

Synergy Award (Johns Hopkins University), Magnetic field vestibular stimulation for rehabilitation, 2015-2017

**SOCIETIES:**

Association for Research in Vision and Ophthalmology (Fellowship 2009)

American Academy of Neurology (Fellowship, 1982)

Bárány Society

Society for Neuroscience

American Neurological Association, 1981

Australian Neuroophthalmology Society (honorary)

Canadian Society of Neurologists (honorary)

Asociacion Colombiana de Neurologia (honorary)

La Société Française de Neurologie (honorary)

Europae Neuro-Ophthalmolgiae Societatis, Fellow

German Neurological Society (full member)

Swiss Neurological Society (corresponding member)

Deutsche Gesellschaft für Klinische Neurophysiologie (DGKN) (honorary member)

Bielschowsky Society (Germany) (honorary member)

Italian Neurology Society (honorary member)

**EDITORIAL BOARDS:**

Investigative Ophthalmology (1977-1980)

Reviews of Oculomotor Research (1982-1994)

Journal of Neuro-Ophthalmology (1982-1997)

Neuro-Ophthalmology (1983-2003), co-managing editor (1990-1992)

Journal of the Neurological Sciences (1984-87)

Journal of Vestibular Research (1989-2002)

European Neurology (1992-1997)

Brain (1994-1997)

Annals of Neurology (1973-1989, 1997-2008), (associate editor 1997 -2005)

The Cerebellum (2001- )

Frontiers of Neuro-otology (2010- ) associate editor

Journal of the Society of Italian Neurology (2011- )

**ADMINISTRATIVE POSITIONS**:

Director: The Johns Hopkins Neurology Residency Selection Committee (1975-1979)

Secretary-Treasurer: Maryland Neurological Society (1976-1980)

Director: The Johns Hopkins Neurology Outpatient Department (1977-1981)

Member, Medical School Council (1979-1980, 1986-88)

Director: Clinical Neurophysiology Laboratories (1982-1993)

Director: Clinical Eye Movement Laboratory (1982-2015)

Member, Public Relations Committee, Society for Neuroscience (1985-88)

Director: Neurology Residency Program (1986-90)

Course Director: American Academy of Neurology, Neuroophthalmology (1986-90), Neurootology (1995-97), Emergency Room Management of Neuroophthalmic Disorders (1993-96), Eye movements and nystagmus (2001-04).

Chair: Johns Hopkins Medical School Associate Professors Promotion Committee (1988-1991)

Member, Otolaryngology (1990) and Ophthalmology (2002) search committees

Member, Scientific Program Committee, American Academy of Neurology (1991-1993)

Member, Medical Advisory Board: Vestibular Disorders Association (VEDA) (1991-2012)

Member, Board of Directors: Society for Progressive Supranuclear Palsy (1992-1997)

Member, Medical Advisory Board: Society for Progressive Supranuclear Palsy (1992-2004)

Advisory panel neuro-otologic assessment tests, Naval Aerospace Medical Research Laboratory, 1992

Chair: Neurology Department Appointment and Promotions Committee (1992-1996)

Chair: Honorary Membership Committee, American Neurological Association (1995-1997)

Chair: Neurology Department Finance Committee (1996-2006)

Course co-director: Vestibular and Auditory Disorders, Johns Hopkins Hospital, March, 1997

Vice-chair for finance, Department of Neurology (1998-2006)

Long range planning committee, American Neurological Association (2002-2004)

Director: The Johns Hopkins Neurology residency selection committee (2005-2007)

Faculty development Committee, American Neurological Association, (2006-08)

Member, Committee on Animal Research, Society of Neuroscience (2007-10)

Chair, National Eye Institute search committee for an ocular motor clinician scientist (2008)

Co-director, Neuroscience teaching course, First year medical students, Johns Hopkins (2010-2017)

**BOARD CERTIFICATION**:

American Board of Psychiatry and Neurology (1976)

**TRAINEES:** 85 postdoctoral fellows, graduate students or visiting scientists have come for a sustained (more than two months) period to work in our laboratory (1975-2017). Those with an asterix are K-award or equivalent trainees.

1975 Takeshi Kasai, (visiting scientist, Bioengineering, Japan)

1976 Atsumi Yamazaki, (postdoc, Ophthalmology, Japan)

1976 Robert Muratore (engineering student, USA)

1978 R. John Leigh (postdoc, Neurology, USA)

1980 William Keys (postdoc, Neurology, USA)

1982 Timothy Hain (postdoc, Neurology USA)*

1983 Bruno Estanol, (visiting scientist, Neurology, Mexico)

1985 Ronald Tusa (resident, postdoc, Neurology, USA)*

1985 Steve Gordon (medical student, resident Ophthalmology, USA)

1986 Tone Suzuki (postdoc, Ophthalmology, Japan)

1986 Michael Fetter (postdoc, Neurology, Germany)

1988 Leah Levi (postdoc (shared), Ophthalmology, University of California, San Diego)

1988 Chiara Straatthof (medical student, Neurology, The Netherlands)

1988 Marina Tijssen (medical student, Neurology, The Netherlands)

1989 Jun-Ru Tian (postdoc, Neurology, China)

1989 William Fletcher (postdoc, Neurology, Canada),

1990 Akihiko Oohira (postdoc, Ophthalmology, Japan)

1990 Jocelyne Ventre (postdoc, Neuropsychology, France)

1990 Helena Papageorgiou, (postdoc, Neurology, Greece)

1990 Stevie Tan (medical student, Ophthalmology, The Netherlands)

1990 Richard Lewis (resident, postdoc, Neurology, USA) *

1991 Caroline Tilikete, (postdoc, Neurology, France)

1991 Jan Ygge (postdoc, Ophthalmology, Sweden)

1992 Bertrand Gaymard (postdoc, France)

1993 Miep Van der Drift, (medical student, The Netherlands)

1993 Philip Kramer (postdoc, Neurology, USA)*

1993 Dominik Straumann (postdoc, Neurology, Switzerland)

1993 Maurizio Versino (postdoc, Neurology, Italy)

1993 Mineo Takagi (postdoc, Ophthalmology, Japan)

1993 Nicolas Perez (visiting scientist, Otolaryngology, Spain)

1994 Elliot Frohman, (postdoc, Neurology, USA)

1994 Tsa-tung Tsai (postdoc, Neurology, Taiwan)

1994 David Solomon (postdoc, Neurology, USA)

1994 Hiro Ichijo (postdoc, Otolaryngology, Japan)

1996 Nate Carter (postdoc, Neurology, USA)

1996 Grace Peng (postdoc, bioengineering, USA)

1996 Choongkil Lee (visiting scientist, Neurophysiology, South Korea)

1997 Mark Walker (postdoc, Neurology, USA)*

1997 Heimo Steffen (postdoc, Ophthalmology, Germany)

1997 Stefan Hegemann (resident, Otolaryngology, Germany)

1998 Naoto Hara (postdoc, Ophthalmology, Japan)

1998 Leah Averbuch-Heller (postdoc, Ophthalmology, Israel)

1998 George McKenna (postdoc, Neurology, USA)

1998 Peter Trillenberg (postdoc, Neurology, Germany)

1998 Vivek Patel (med student, Ophthalmology, Canada)

1998 Matt Taylor (med student, psychiatry, England)

2000 Sharon Tow (postdoc (shared), Ophthalmology, USA)

2000 Luis Mejico (postdoc (shared), Neurology, USA)

2001 David Newman-Toker (postdoc, Neurology, USA)*

2001 Majid Fotuhi (resident, Neurology, USA)

2001 Scott Eggers (postdoc, Neurology, USA)

2001 Alessandra Rufa (visiting scientist, Neurology, Italy)

2002 Stefano Ramat, (postdoc, bioengineering, Italy)

2002 Sarah Ying (postdoc, Neurology, USA)*

2002 Nicholas de Pennington (medical student, Cambridge, England)

2003 Elena Isotalo (postdoc, Otolaryngology, Finland)

2003 Jing Tian (postdoc, Bioengineering, USA)

2004 Xioyan Shan (postdoc, Ophthalmology, USA)

2005 Aasef Shaikh (postdoc, Neurology, USA)

2005 Anne-Catherine Huys (medical student, Oxford, England)

2006 Alex Tarnutzer (postdoc, Neurology, Switzerland)

2007 Haiyin Chen-Harris (graduate student, shared with Reza Shadmehr, Bioengineering, USA)

2007 Vincent Ethier (graduate student, shared with Reza Shadmehr, Bioengineering, Canada)

2008 Minnan Xu-Wilson (graduate student, shared with Reza Shadmehr, Bioengineering, USA)

2008 Masahiko Fujita (visiting scientist, Bioengineering, Japan)

2008 Nihal Sinha (medical student, Cambridge, England)

2008 Naoir Zaher (postdoc, Neurology, Syria)

2009 Amir Kheradmand (postdoc, Neurology, USA)*

2009 Howard Ying (Wilmer training grant)”

2009 Ichiro Hamasaki (postdoc, Ophthalmology, Japan)

2010 Tammy Ng (medical student, Neurology, England)

2011 Wu Choi (medical student, Neurology, Canada)

2011 Xu Xiang Zhang (postdoc, shared David Newman-Toker, Neurology, China)

2011 Dan Gold (resident, Neurology, USA)

2012 Yuri Agrawal (faculty, Johns Hopkins)*

2013 Seung-Han Lee (postdoc, shared with David Newman-Toker, Neurology, South Korea)

2013 Prem Jareonsettasen (medical student, Cambridge, England)

2013 Jorge Otero-Millan (postdoc, bioengineer, Spain/USA)*

2014 Saverio Silipo (resident, Neurology, Italy)

2015 Ayse Ilksen Colpak (postdoc, shared with Amir Kheradmand, Neurology, Turkey)

2017 Francesco Fornisari (medical student, Milan, Italy)

2017 Nicolas Perez (visiting scientist, Pamplona, Spain)

2017 Chang Tzu-Pu (postdoc, shared with David Newman-Toker, Neurology, Taiwan)

2017 Roksolyana Tourkevich (postdoc, shared with Dan Gold, Neurology, USA)

2017 Ram Narayan (postdoc, shared with neuroimmunology)

**INTERVIEWS AND VIDEO CONTRBIUTIONS:**

Article in Washington Post about diagnosis of medical mysteries:

<https://www.washingtonpost.com/national/health-science/doctors-puzzled-by-womans-dizziness-and-amplified-body-sounds/2014/11/24/200dcc22-593b-11e4-8264-deed989ae9a2_story.html>

Interview: University of California TV:

<https://player.fm/series/health-and-medicine-audio/the-eyes-have-it-a-interview-with-david-zee-md>

https://www.youtube.com/watch?v=6GjGcUlwbAA

Interview: Belgrade Balance Forum

<https://youtu.be/vMOhMYkhlPA>

Interview: University of Utah

https://collections.lib.utah.edu/details?id=1278469

Video Library Contributions

Daniel R. Gold, MD and David S. Zee, MD. Range of Eye Movements and Evaluation for Nystagmus. PDF. [NOVEL]. 2016. Available at: http://content.lib.utah.edu/cdm/ref/collection/EHSL-NOVEL/id/2342. April 28, 2016.

Daniel R. Gold, MD and David S. Zee, MD. VOR (Slow and Fast). PDF. [NOVEL]. 2016. Available at: http://content.lib.utah.edu/cdm/ref/collection/EHSL-NOVEL/id/2348. April 28, 2016.

Daniel R. Gold, MD and David S. Zee, MD. Convergence. PDF. [NOVEL]. 2016. Available at: http://content.lib.utah.edu/cdm/ref/collection/EHSL-NOVEL/id/2341. April 28, 2016.

Daniel R. Gold, MD and David S. Zee, MD. Smooth Pursuit. PDF. [NOVEL]. 2016. Available at: http://content.lib.utah.edu/cdm/ref/collection/EHSL-NOVEL/id/2343. April 28, 2016.

**BIBLIOGRAPHY (OVER 470 PUBLICATIONS EXCLUDING ABSTRACTS)**

[**Google Scholar**](https://scholar.google.com/citations?user=-cOeGw0AAAAJ&hl=en)

[**Pubmed Citations**](http://www.ncbi.nlm.nih.gov/pubmed?term=(ZEE%20D%5bAuthor%5dAND%20Hopkins%5bAD%5d)OR%20ZEE%20DS%5bAuthor%5d)

**CITATION INDEX (Google Scholar: 24683 citations, h-index 72, i10-index 293)**

**MOST VIEWED: Kim, JS Zee, DS, Benign Paroxysmal Positional Vertigo, N. Engl. J. Med, 370: 1138-47, 2014. 96056 VIEWS (Top 20% of all NEJM articles)**

**1968**

**Zee**, D.S. and Zinkham, W., Malate dehydrogenase in *ascaris suum*: Characterization, ontogeny, and genetic control. Arch. Biochem. Biophys. 126:574-584, 1968.

**1970**

**Zee**, D.S., Isenee, H. and Zinkham, W., Polymorphism of malate dehydrogenase in *ascaris suum*. Biochem. Genet. 4:253-157, 1970.

**1974**

**Zee**, D.S., Freeman, J.M. and Holtzman, N.A., Ophthalmoplegia in maple syrup urine disease. J. Pediatrics, 84:113-115, 1974.

**Zee**, D.S., Friendlich, A. and Robinson, D.A., The mechanism of downbeat nystagmus. Arch. Neurol. 30:227-237, 1974.

**Zee**, D.S., Griffin, J. and Price, D.L., Unilateral pupillary dilatation during adversive seizures. Arch. Neurol., 30:403-405, 1974.

Engel, W.K, and **Zee,** D.S. Letter: Acupuncture myopathy? (Remembrance of things passed). N Engl J Med., 291:801, 1974.

**1975**

**Zee**, D.S. and Zinkham, W., Demonstration of parasitism in tissues by electrophoretic definition of parasite enzymes in host tissues. J. Pediatrics, 86:408-410, 1975.

**Zee**, D.S., Cogan, D.G., Robinson, D.A. and Engel, W.K., Analysis of eye movements in members of a family with familial late-onset cerebellar ataxia. TTTTTTransactions of the American Neurological Association, 100:98-103, 1975.

**1976**

**Zee**, D.S., Optican, L., Cook, J.D., Robinson, D.A. and Engel, W.K., Slow saccades in spinocerebellar degeneration. Arch. Neurol., 33:243-251, 1976.

Yee, R.D., Cogan, D.G. and **Zee**, D.S., Ophthalmoplegia and dissociated nystagmus in abetalipoproteinemia. Arch. Ophthalmol., 94:571-575, 1976.

**Zee**, D.S., "Dizziness and Vertigo" Chapter 128. In: Harvey, et al., PPPPrinciples and Practice of MedicineP, Appleton-Century-Crofts, 19th Edition, New York, 1976.

**Zee**, D.S., "Disorders of the Brain Stem". Chapter 129. In: Harvey, et al., Principles and Practice of Medicine, Appleton-Century-Crofts, 19th Edition, New York, 1976.

**Zee**, D.S., Yee, R.D., Cogan, D.G., Robinson, D.A. and Engel, W.K., Ocular motor abnormalities in hereditary cerebellar ataxia. Brain, 99:207-234, 1976.

Yee, R.D., Trese, M., **Zee**, D.S. and Cogan, D.G., Ocular manifestations of acute pandysautonomia. Amer. J. Ophthalmol., 81:740-744, 1976.

**Zee**, D.S., Yee, R.D. and Robinson, D.A., Optokinetic responses in labyrinthine-defective human beings. Brain Res., 113:423-428, 1976.

Yee, R.D., Cogan, D.G., **Zee**, D.S., Baloh, R. and Honrubia, V., Rapid eye movements in myasthenia gravis. II. Electro-oculographic analysis. Arch Ophthalmol., 94:1465-1472, 1976.

**1977**

**Zee**, D.S. and Yee, R.D., Abnormal saccades in paralytic strabismus. Amer. J. Ophthalmol., 83:112-114., 1977.

**Zee**, D.S., Suppression of vestibular nystagmus. Ann Neurol., 1:207, 1977.

**Zee**, D.S., Disorders of eye-head coordination. In: Eye Movements, eds. B.A. Brooks and F.J. Bajandas, Plenum Press, New York, pp. 9-29, 1977.

**Zee**, D.S., Yee, R.D. and Singer, H.S., Congenital ocular motor apraxia. Brain, 100:581-589, 1977.

**1978**

Kasai, T. and **Zee**, D.S., Eye-head coordination in labyrinthine-defective human beings. Brain Res. 144:123-141, 1978.

**Zee**, D.S., Ophthalmoscopy in the evaluation of vestibular disorders. Ann Neurol., 3:373-374, 1978.

**Zee**, D.S., The organization of the brain stem ocular motor subnuclei. Ann Neurol., 4:384-385, 1978.

**1979**

Muratore, R. and **Zee**, D.S., Pursuit after-nystagmus. Vision Research, 19:1057-1059, 1979.

**Zee**, D.S. and Robinson, D.A., A hypothetical explanation of saccadic oscillations. Ann. Neurol. 5:405-414, 1979.

Yamazaki, A. and **Zee**, D.S., Rebound nystagmus: an electro-oculographic analysis of a case with a floccular tumor. Brit J Ophthalmol., 63:782-786, 1979.

**Zee**, D.S. and Robinson, D.A., Clinical applications of oculomotor models. In: Topics in Neuro-Ophthalmology, Ed. H.S. Thompson, Williams & Wilkens, Baltimore, pp. 266-285. 1979.

**1980**

**Zee**, D.S., Leigh, R.J. and Mathieu-Millaire, F., Cerebellar control of ocular gaze stability. Ann. Neurol., 7:37-40, 1980.

**Zee**, D.S., "Dizziness, vertigo and hearing loss" Chapter 119. In: Harvey, et al., Principles and Practice of Medicine, Appleton-Century- Crofts, 20th Edition, New York. 1980.

**Zee**, D.S., "Brain stem dysfunction". Chapter 127. In: Harvey, et al., Principles and Practice of Medicine, Appleton-Century- Crofts, 20th Edition, New York, 1980.

Leigh, R.J. and **Zee**, D.S., Eye movements of the blind. Invest. Ophthalmol. 19:327-331, 1980.

**Zee**, D.S., Ocular motor control. In: Neuro-Ophthalmology Vol. 1, Eds. S. Lessell and J.W.T. van Dalen, Excerpta Medica, Amsterdam, pp. 131-145, 1980.

Halmagyi, G., Rudge, P., Gresty, M., Leigh, R.J. and **Zee**, D.S., Treatment of periodic alternating nystagmus. Ann. Neurol., 8:609-611, 1980.

**1981**

**Zee**, D.S., The vestibulo-ocular reflex: clinical concepts. In: Models of Ocular Motor Behavior and Control, Ed. B. Zuber, CRC Press, Long Beach, California, pp. 257-278, 1981.

Leigh, R.J., Robinson, D.A. and **Zee**, D.S., A hypothetical explanation of periodic alternating nystagmus: instability in the optokinetic-vestibular system. Ann. N. Y. Acad. Sci ., 374:619-635, 1981.

Robinson, D.A. and **Zee**, D.S., Theoretical considerations of the function and circuitry of various rapid eye movements. In: Progress in Ocular Motor Research, Eds. W. Becker and A. Fuchs, Elsevier, Vol. 12, pp. 3-11, 1981.

Gourdeau, A., Miller, N.R., **Zee**, D.S. and Morris, J., Central ocular motor abnormalities in Duane's retraction syndrome. Arch. Ophthalmol. 99:1809-1810, 1981.

**Zee**, D.S., Yamazaki, A., Butler, P.H. and Gücer, G., Effects of ablation of the flocculus and paraflocculus on eye movements in primate. J. Neurophysiol., 46:878-899, 1981.

Pulaski, P.D., **Zee**, D.S. and Robinson, D.A., The behavior of the vestibulo-ocular reflex at high velocities of head movement. Brain Res.. 22:159-165, 1981.

**1982**

**Zee**, D.S., Cerebellar control of eye movements. In: Nystagmus and Vertigo: Clinical Approaches to the Patient with Dizziness, Eds. V. Honrubia and M. Brazier, Academic Press, pp. 241-249, 1982.

Leigh, R.J., Newman, S.A., **Zee**, D.S. and Miller, N.R., Visual following during stimulation of an immobile eye (The open loop condition). Vision Research, 22:1193-1197, 1982.

**Zee**, D.S., Ocular motor control: the cerebellum. In: Neuro-Ophthalmology, Vol. II, Eds. S. Lessell and J.T.W. van Dalen, Excerpta Medica, Amsterdam, pp. 136-147, 1982.

**Zee**, D.S. Ocular motor abnormalities related to lesions in the vestibulocerebellum in primate. In: Functional Basis of Ocular Motility Disorders, Eds. G. Lennerstrand, D.S. Zee and E.L. Keller, Pergamon Press, Oxford, pp. 423-430, 1982.

**Zee**, D.S., Preziosi, T. and Proctor, L.,Bechterew's phenomenon in a human patient. Ann. Neurol. 12:495-496, 1982.

Leigh, R.J. and **Zee**, D.S., The diagnostic value of abnormal eye movements: a pathophysiological approach. The Johns Hopkins Medical Journal, 151:122-135, 1982.

Lennerstrand, G., **Zee**, D.S. and Keller, E.L., Eds. Functional Basis of Ocular Motility Disorders, Pergamon Press, Oxford, 1982.

**Zee**, D.S., Butler, P.H., Optican, L.M., Tusa, R.J. and Gücer, G., Effects of bilateral occipital lobectomies on eye movements in monkeys: preliminary observations. In: Physiological and Pathological Aspects of Eye Movements, Eds. A. Roucoux and M. Crommelinck, The Hague, Dr. W. Junk, pp. 225-232, 1982.

**1983**

**Zee**, D.S. and Leigh, R.J., The neural control of eye movements. In: Clinical Neurosciences, Ed. R. Rosenberg, Churchill-Livingstone, New York, Vol. 5, pp. 519-546, 1983.

Leigh, R.J. and **Zee**, D.S., The Neurology of Eye Movements, Contemporary Neurology Series, F.A. Davis, Philadelphia, 1983.

**Zee**, D.S., Nystagmus in multiple sclerosis. Bull. Soc. Belge Ophal.. 208-I: 211-217, 1983.

**Zee**, D.S., Chu, F.C., Leigh, R.J., Savino, P.T., Schatz, N.J., Reingold, D.B. and Cogan, D.G., Blink-saccade synkinesis. Neurology, 33:1233-1236, 1983.

**Zee**, D.S. and Leigh, R.J., Disorders of eye movements. In: Neuro-Ophthalmology, Neurological Clinics of North America, Eds. C. Smith and R. Beck, Saunders, Vol. 1, pp. 909-928, 1983.

Hirst, L.W., Clark, A.W., Wolinsky, J.S., **Zee**, D.S., Kaizer, H., Miller, N.R., Tutschka, P.J. and Santos, G.W., Downbeat nystagmus -- A case report of herpetic brain stem encephalitis. J. Clin. Neuro-Ophthalmol. 3:245-249, 1983.

**1984**

**Zee**, D.S. and Optican, L.M., Mechanisms of ocular oscillations. In: Tremor, Eds. L. Findley and R. Capildeo, MacMillan Press, Ltd., London, 1984.

Demer, J. and **Zee**, D.S., Vestibulo-ocular and optokinetic deficits in albinos with congenital nystagmus. Invest. Ophthalmol. Vision. Sci., 25:739-745, 1984.

**Zee**, D.S., New concepts of cerebellar control of eye movements. Otolaryngology-Head and Neck Surgery, 92:59-62, 1984.

Leigh, R.J. and **Zee**, D.S., Episodic vertigo. In: Conn's Current Therapy, 36th Edition, pp. 722-728, 1984.

**Zee**, D.S. and Leigh, R.J., Brain stem dysfunction. In: Principles and Practice of Medicine Eds. A.M. Harvey, R.J. Johns, V.A. McKusick, A.H. Owens and R.S. Ross, 21st Edition, Appleton-Century-Crofts, New York, pp. 1242-1249, 1984.

**Zee**, D.S., Dizziness, vertigo and hearing loss. In: Principles and Practice of Medicine Eds. A.M. Harvey, R.J. Johns, V.A. McKusick, A.H. Owens, R.S. Ross, 21st Edition, Appleton-Century-Crofts, New York, pp. 1249-1254, 1984.

**Zee**, D.S., Ocular motor control: cerebral control of saccadic eye movements. In: Neuro-Ophthalmology, Vol. III, Eds. S. Lessell and J.T.W. van Dalen, Excerpta Medica, Amsterdam, pp. 141-156, 1984.

Optican, L.M. and **Zee**, D.S., A hypothetical explanation of congenital nystagmus. Biological Cybernetics, 50:119-134, 1984.

Lisberger, S.G., Miles, F.A. and **Zee**, D.S., Signals used to computer errors in the monkey vestibulo-ocular reflex: possible role of the flocculus. J. Neurophysiol. 52:1140-1153, 1984.

**Zee**, D.S., Chu, F.C., Optican, L.M. and Carl, J.R., Graphical analysis of paralytic strabismus using the Lancaster red-green test. Am. J. Ophthalmol., 97:587-592, 1984.

**Zee**, D.S., Treatment of vertigo. In: Current Therapy in Internal Medicine , Eds. T. Bayless, M.C. Brain and R.W. Cherniack, B.C. Decker, Inc., Toronto, 1265-1269, 1984.

Robinson, D.A., **Zee**, D.S., Hain, T.C., Holmes, A.M. and Rosenberg, L.F.: Alexander's law -- Its behavior and origin in the human vestibulo-ocular reflex. Ann. Neurol., 16:714-722, 1984.

**1985**

**Zee**, D.S. and Optican, L.M., Studies of adaptation in human ocular motor disorders. In: Reviews of Oculomotor Research Adaptive Mechanisms in Gaze Control: Facts and Theories, Vol. 1, Eds. A. Berthoz and G. Melvill Jones, Elsevier, pp. 165-176, 1985.

**Zee**, D.S., Vertigo. In: Current Therapy in Neurologic Disease, Ed. R.T. Johnson, B.C. Decker, Inc., 8-13, 1985.

**Zee**, D.S. and Leigh, R.J., Eye movement control. In: Scientific Basis of Clinical Neurology, Eds. M. Swash and C. Kennard, Churchill- Livingstone, Edinburgh, pp. 133-149, 1985.

Optican, L.M., **Zee**, D.S. and Chu, F.C., Adaptive response to ocular muscle weakness in human pursuit and saccadic eye movements. J. Neurophysiol., 54:110-122, 1985.

**Zee**, D.S., Perspectives on the pharmacotherapy of vertigo. Arch. Otolaryngol., 111:609-612, 1985.

Carl, J.R., Optican, L.M., Chu, F.C. and **Zee**, D.S., Head shaking and vestibulo-ocular reflex in congenital nystagmus. Invest Ophthalmol Vision Sci. 26:1043-1050, 1985.

Cannon, S.C., Leigh, R.J., **Zee**, D.S. and Abel, L., The effect of the rotational magnification of corrective spectacles on the quantitative evaluation of the VOR. Acta Otolaryngol., (Stockh), 100:81-88, 1985.

**Zee**, D.S., Mechanisms of nystagmus. Amer. J. Otol, Suppl. 30-34, 1985.

**1986**

Hain, T.C., **Zee**, D.S. and Mordes, M., Blink-induced saccadic oscillations, Annals of Neurology,19:299-301, 1986.

**Zee**, D.S., Examination of eye movements in the diagnosis of diseases of the vestibular system. In: Otolaryngology-Head and Neck Surgery Eds. C.W. Cummings, et al., C. V. Mosby, St. Louis, pp. 2765-2778, 1986.

**Zee**, D.S., Tusa, R.J., Herdman, S.J., Butler, P.H. and Gücer, G., The acute and chronic effects of bilateral occipital lobectomy upon eye movements in monkey. In: Adaptive Processes in Visual and Oculomotor Systems, Eds. E. Keller and D.S. **Zee**, Pergamon, Oxford, pp. 267-274, 1986.

Tusa, R.J., **Zee**, D.S. and Herdman, S.J., Recovery of oculomotor function in monkeys with large unilateral cortical lesions. In: Adaptive Processes in Visual and Oculomotor Systems, Eds. E. Keller and D.S. Zee, Pergamon, Oxford, pp. 209-216, 1986.

Keller, E. and **Zee**, D.S. editors, Adaptive Processes in Visual and Oculomotor Systems, Pergamon, Oxford, 1986.

Keller, E. and **Zee**, D.S., Summary of symposium on adaptive processes in visual and oculomotor systems. Binocular Vision, 1:171-173, 1986.

**Zee**, D.S. and Hain, T.C. Adaptation to ocular motor disorders: clinical implications. In: Sensorimotor Plasticity: Theoretical, Experimental and Clinical Aspects, eds. Ron, S., Schmid, R. and Jeannerod, M., Inserm, Paris, pp. 101-120, 1986.

**Zee**, D.S., Oculomotor control. In: Diseases of the Nervous System eds. Asbury, A.K., McKhann, G.M., and McDonald, W.I., W.B. Saunders, Philadelphia, 507-519, 1986.

Fetter, M., Hain, T.C., and **Zee**, D.S., Influence of eye and head position on the vestibulo-ocular reflex. Exp. Brain Res.,64:208-216, 1986.

Tusa, R.J., **Zee**, D.S. and Herdman, S.J., Effect of unilateral cerebral hemispheral lesions on eye movements in monkeys: Saccades and quick phases. J. Neurophysiol 56:1590-1626, 1986.

Optican, L.M., **Zee**, D.S., and Miles, F.A., Floccular lesions abolish adaptive control of postsaccadic ocular drift in primates. Exp. Brain Res. 64:596-598, 1986.

**Zee**, D.S., Brain stem and cerebellar deficits in eye movement control. Trans. Ophthalmol. Soc. UK., 105:599, 1986.

**1987**

Kapoula, Z., Hain, T.C., **Zee**, D.S. and Robinson, D.A., Adaptive changes in post-saccadic drift induced by patching one eye. Vision Research, 27:1299-1307, 1987.

**Zee**, D.S., Hain, T.C. and Carl J.R., Abduction nystagmus in internuclear ophthalmoplegia. Ann. Neurol, 21:383-388, 1987.

Hain, TC, Fetter, M. and **Zee**, D.S., Head shaking nystagmus in patients with unilateral peripheral vestibular lesions. Am. J. Otolaryngol. 8:36-47, 1987.

**Zee**, D.S., Eye movement disorders in cerebellar disease. ENG Report, (ICS Medical), March issue, 1987.

Lasker, A.G., **Zee**, D.S., Hain, T.C., Folstein, S., Singer, H., Saccades in Huntington's Disease: Initiation defects and distractibility. Neurology, 37: 36-370, 1987.

Berthoz, A., Israel, I., Vieville, T. and **Zee**, D.S., Linear head displacement measured by the otoliths can be reproduced through the saccadic system, Neuroscience Letters 82:285-290, 1987.

**Zee**, D.S., Tusa, R., Herdman, S., Butler, P., and Gücer, G., Effects of occipital lobectomy upon eye movements in primate. J. Neurophysiol., 58:883-907, 1987.

Hain, T.C., **Zee**, D.S. and Maria, B., Modification of the dynamics of the vestibuloocular reflex by head tilt in patients with cerebellar lesions. In: The Vestibular System Neurophysiologic and Clinical Research eds. M.D. Graham and J.L. Kemink, Raven Press, New York, pp. 217-223, 1987.

Sanders, E.A., de Keizer, R.J., and **Zee**, D.S., eds. Eye Movement Disorders, Dr. W. Junk Publishers, Dordrecht, 1987.

**Zee**, D.S., Disorders of horizontal and vertical gaze. In: Eye Movement Disorders, Eds. E.A. Sanders, R.J. de Keizer, and D.S. **Zee**, Dr. W. Junk Publishers, Dordrecht, 173-182, 1987.

Cannon, S. and **Zee**, D.S., El integrado neural del sistema oculomotor. In: Fisiologia y Fisiopatologia del Sistema Oculomotor. editors, Gavilan, C. and Gavilan, J., Ediotoria Garci, Madrid, pp. 32-42, 1987.

Moses III, H. and **Zee**, D.S.: Multi-infarct PSP. Neurology,37:1819 (letter), 1987.

**1988**

Cannon, S.C. and **Zee**, D.S., The neural integrator of the oculomotor system. In: Current Neuro-ophthalmology, Ed. S. Lessell and J.T.W. van Dalen, Vol. 1, Year Book Medical Publishers, 123-138, 1988.

**Zee**, D.S., New concepts of vestibular nystagmus. In: Vestibular Disorders, eds., H. Barber and J. Sharpe, Year Book Medical Publishers, Chicago, 189-200, 1988.

**Zee**, D.S. The management of patients with vestibular disorders. In: Vestibular disorders, eds., H. Barber and J. Sharpe, Year Book Medical Publishers, Chicago, 254-274, 1988.

Berthoz, A., Israel, I., Vieville, T. and **Zee**, D.S., Linear displacement can be derived from otolithic information and stored on spatial maps controlling the saccadic system. Adv. in Oto-Rhino-Laryngol.,42:76-82, 1988.

**Zee**, D.S. and Proctor, L.R., Dizziness, vertigo and hearing loss. In: Principles and Practice of Medicine, Appleton, pp. 1039-1042, 1988.

**Zee**, D.S., Brain stem dysfunction. In: Principles and Practice of Medicine, Appleton, pp. 1033-1038, 1988.

Hain, T.C., **Zee**, D.S. and Maria, B., Tilt-suppression of the vestibulo-ocular reflex in patients with cerebellar lesions. Acta Otolaryngol. 105: 13-20, 1988.

Fetter, M., and **Zee**, D.S., Recovery from unilateral labyrinthectomy in the Rhesus monkey, J. Neurophysiol. 59:370-393, 1988.

Fetter, M., **Zee**, D.S., and Proctor, L.R., Effects of lack of vision and of occipital lobectomy upon recovery from unilateral labyrinthectomy in the Rhesus monkey. J. Neurophysiol.,59: 394-407, 1988.

Lasker, A.G., **Zee**, D.S., Hain, T.C., Folstein, S.E. and Singer, H. Saccades in Huntington's disease: slowing and dysmetria. Neurology, 38:427-431, 1988.

Levin, S, Luebke, A., Hain, T.C., **Zee**, D.S., Robinson, D.A. and Holzman, P.S., Smooth pursuit eye movements in schizophrenics: quantitative measurements with the search-coil technique, J. Psych. Research, 22:195-206, 1988.

**Zee**, D.S., Fetter, M. and Proctor, L., Recovery from unilateral labyrinthectomy in primate: effects of visual inputs and considerations upon Ewald’s second law. In: Basic and Applied Aspects of Vestibular Function, J.C. Huang, N.G. Daunton, and V. J. Wilson, eds., University of Hong Kong Press, 125-132, 1988.

Fetter, M. and **Zee**, D.S., Recovery from unilateral labyrinthectomy in primate. In: Post-lesion Neural Plasticity, ed. H. Flohr, Springer-Verlag, 305-312, 1988.

**Zee**, D.S., Ocular motor and vestibular disorders. In: Experimental Surgery and Physiology: Induced Animals Models of Human Disease, eds. Swindle, M.M., Adams, R.J., Williams and Wilkins, Baltimore, pp. 264-267, 1988.

**Zee,** D.S., Recovery from unilateral labyrinthectomy in Rhesus monkey. Equil*.* Res. 47:18-21, 1988.

**1989**

Buettner, U. and **Zee**, D.S., Vestibular testing in comatose patients, Arch. Neurol. 46:561-563, 1989.

**Zee**, D.S. and Levi, L., Neurological aspects of vergence eye movements, Revue Neurologique 145 613-620, 1989.

Kelly, B.J., Rosenberg, M., **Zee**, D.S. and Optican L.M., Unilateral pursuit-induced congenital nystagmus. Neurology, 39:414-416, 1989.

**Zee**, D.S., Adaptation and the ocular motor system. Bull. Soc. Belge Ophthalmol., 237:191-207, 1989.

Hain, T.C. and **Zee**, D.S., Vergence. Bull. Soc Belge Ophthalmol. 237:145-161, 1989.

Tijssen, M., Straathof, C., Hain, T.C. and **Zee**, D.S., Optokinetic afternystagmus in humans: normal values of amplitude, time constant and asymmetry. Annals Otol., Rhinol. Laryngol. 98:741-746, 1989.

**Zee**, D.S., Fetter, M., and Proctor, L.R., Compensation for unilateral labyrinthectomy in normal and occipital-lobectomized monkeys. La Revue D' ONO, 25:55-59, 1989.

**Zee**, D.S., Eye movement disorders and ocular motor control. In: Current Opinion in Neurology and Neurosurgery, ed. D. Burke, Current Science Ltd, 2, 749-758, 1989.

**Zee**, D.S., The place for vestibular function tests. The Western Journal of Medicine, 150:81-82 (editorial), 1989.

Tusa, R.J. and **Zee**, D.S., Cerebral control of smooth pursuit and optokinetic nystagmus. In: S. Lessell and J.T.W. van Dalen, Current Neuro-ophthalmology, Year Book, Chicago, 115-146, 1989.

**Zee**, D.S., Fetter, M., and Proctor, L.R., Recovery from unilateral labyrinthectomy in normal and occipital lobectomized monkeys. In Vestibular Compensation: Facts, Theories and Clinical Perspectives, eds., Lacour, M., Toupet, M., Denise, P., and Christen, Y., Elsevier, Paris, pp. 73-81, 1989.

**1990**

Fletcher, W., Hain, T.C., and **Zee**, D.S., Optokinetic nystagmus and afternystagmus in human beings: relationship to processing of information about retinal slip. Exp Brain Res, 81: 46-52, 1990.

Fetter, M., **Zee**, D.S., Koenig, E. and Dichgans, J., Head-shaking nystagmus during vestibular compensation in humans and rhesus monkeys. Acta Otolaryngol, 110:175-181, 1990.

Hain, T.C. and **Zee**, D.S., Vergence. In: Neurological Organization of Ocular Movement eds. R. Daroff and A. Neetens, Kugler and Ghedini, Amsterdam, pp. 145-161, 1990.

**1991**

Ashe, J., Hain, T.C., **Zee**, D.S. and Schatz, N.J., Microsaccadic flutter. Brain, 114:461-472, 1991.

Maas, E.F., Ashe, J., Spiegel, P.S., **Zee**, D.S. and Leigh, R.J., Acquired pendular nystagmus in toluene addiction. Neurology, 41:282-285, 1991.

Oohira, A., **Zee**, D.S. and Guyton, D.L., Disconjugate adaptation to long-standing, large-amplitude spectacle-corrected anisometropia. Invest Ophthal Vis Sci., 32:1693-1703, 1991.

Leigh, R.J. and **Zee**, D.S., The Neurology of Eye Movements, F.A. Davis, Philadelphia, Second Edition, 1991.

Tian, J.R., Herdman, S.J., **Zee**, D.S., and Folstein, S., Postural control in Huntington's Disease (HD). Acta Otolaryngol Suppl 481: 333-336, 1991.

**Zee**, D.S., Adaptation to vestibular disturbances: some clinical implications. Neuro-ophthalmology, 11:111-116, 1991.

Hain, T.C. and **Zee**, D.S., Abolition of optokinetic afternystagmus by aminoglycoside ototoxicity. Annals Otol Rhino Laryngol. 100:580-583, 1991.

Tian, J.R, **Zee**, D.S., Lasker, A.G. and Folstein, S., Saccades in Huntington's disease: Predictive tracking and interaction between release of fixation and initiation of saccades. Neurology,41:875-881, 1991.

Leigh, R.J. and **Zee**, D.S., Oculomotor disorders. In: R.H.S. Carpenter, Vision and Visual Dysfunction, Vol. 8., MacMillan, London, pp. 297-319, 1991.

**Zee**, D.S., Eye movements in neurological diagnosis: Huntington's disease and congenital nystagmus, Oculomotor Control and Cognitive Processes: Normal and Pathological Aspects,eds. Zambarbierri, D, and Schmid, R., Elsevier Science Publishers, North-Holland, 9-17, 1991.

Kaminski, H.J., Leigh, R.J., **Zee**, D.S., and Mendez, M.F., Ocular flutter and ataxia associated with AIDS-related complex. Neuro-ophthalmology, 11:163-167, 1991.

Tusa, R.J. and **Zee**, D.S., Development and maintenance of ocular alignment. In: S. Lessell and J.T.W. van Dalen, Current Neuro-ophthalmology, Year Book, Chicago, pp. 129-152, 1991.

**Zee**, D.S., Adaptation to vestibular disturbances: Some clinical implications. Acta Neurol. Belg. 91:97-104, 1991.

**1992**

**Zee**, D.S. and Hain, T.C., Clinical implications of otolith-ocular reflexes. Amer. J. Otol., 13:152-57 1992.

Leigh, R.J. and **Zee**, D.S., Ocular motor control -- normal and abnormal. In: Diseases of the Nervous System, eds., Asbury, A., Mckhann, G.M. and McDonald, W.I., Saunders, pp. 368-388, 1992.

**Zee**, D.S. and Leigh, R.J., Examination of eye movements in the diagnosis of diseases of the vestibular system. In: Otolaryngology- Head and Neck Surgery Eds. C.W. Cummings, et al., C.V. Mosby, St. Louis, 2683-2697, 1992.

Oohira, A. and **Zee**, D.S., Disconjugate ocular motor adaptation in rhesus monkey. Vision Res., 32:489-497, 1992.

Tan, H.S., Shelhamer, M. and **Zee**, D.S., Effect of head orientation and position on VOR adaptation. Ann. N.Y. Acad. Sci .656:158-165, 1992.

Tian, J-R, Herdman, S.J., **Zee**, D.S. and Folstein, S.E., Postural stability in patients with Huntington's disease. Neurology, 42:1232-1238, 1992.

**Zee**, D.S., Internuclear ophthalmoplegia: Pathophysiology and diagnosis. Baillière's Clinical Neurology, 1:455-470, 1992.

Tusa, R.J., **Zee**, D.S., Hain, T.C. and Simonsz, H.J., Voluntary control of congenital nystagmus. Clinical Visual Science, 7:195-210, 1992.

Hain, T.C. and **Zee**, D.S., The dizzy patient: diagnostic approaches. In: Challenges in Neurology, ed. Hachinski, V., F.A. Davis, Philadelphia, pp. 3-14, 1992.

Oohira, A. and **Zee**, D.S., Disconjugate ocular motor adaptation in rhesus monkey. In: Vestibular and Brain Stem Control of Eye, Head and Body Movements, eds. Shimazu, H. and Shinoda, Y., Japan Scientific Societies Press, Hongo, pp. 427-437, 1992.

Ventre, J., **Zee**, D.S., Papageorgiou, H. and Reich, S., Abnormalities of predictive saccades in hemiparkinson's disease (HPD). Brain, 115:1147-1165, 1992.

Hain, T.C. and **Zee**, D.S., Velocity storage in labyrinthine disorders. Ann. N.Y. Acad. Sci., 656:297-304, 1992.

**Zee**, D.S., FitzGibbon, E. and Optican, L.M., Saccade-vergence interactions in human beings. J. Neurophysiol., 68:1624-1641, 1992.

**1993**

Kommerell, and **Zee**, D.S., Latent nystagmus: release and suppression at will. Invest. Ophthal. Vis. Sci., 34:1785-1792, 1993.

Herdman, S.J., Tusa, R.J., **Zee**, D.S., Proctor, L.R., and Mattox, D.E., Single treatment approaches to benign paroxysmal positional vertigo. Arch. Otolaryngol. Head and Neck Surgery, 119:450-454, 1993.

Tiliket, C., Shelhamer, M., Tan, H.S., and **Zee**, D.S., Adaptation of the vestibuloocular reflex with the head in different orientations and positions relative to the axis of body rotation. J. Vestib. Research, 3:181-196, 1993.

**Zee**, D.S. and Hain, T.C., Otolith-ocular reflexes. In: The Vestibulo-Ocular Reflex and Vertigo, eds. J.A. Sharpe and H.O. Barber, Raven Press, New York, pp. 69-78, 1993.

Rothlind, J.C., Brandt, J., **Zee**, D., Codori, A.M. and Folstein, S., Unimpaired verbal memory and oculomotor control in asymptomatic adults with the genetic marker for Huntington's disease. Arch. Neurol 50:799-802, 1993.

**Zee**, D.S., Afternystagmus and headshaking nystagmus. Equilibrium Research, 52:442-447, 1993.

Gauthier, G.M., Vercher, J-L., and **Zee,** D.S., Modification de l'alignement oculaire chez l'homme a la suite d'une deviation passive prolongee d'un oeil. La Revue d'ONO 23/24, 16-21, 1993.

Lewis, R.F. and **Zee,** D.S., Abnormal spatial localization with trigeminal-oculomotor synkinesis: evidence for a proprioceptive effect. Brain, 116:1105-1118, 1993.

Lewis, R.F. and **Zee,** D.S., Ocular motor disorders associated with cerebellar lesions: pathophysiology and topical localization. Rev. Neurol . (Paris), 149:665-677, 1993.

Shelhamer, M., **Zee,** D.S. and Herdman, S. Saccade trajectories with different head orientations. In Proceedings of the XVth Bárány Society Meeting, eds. Krejcova, H., Jerabek, J., CNEXIM, Prague, pp. 122-126, 1993.

Fetter, M., Tweed, D., Misslisch, D., Fischer, D., **Zee,** D.S., Koenig, E. and Dichgans, J., Three-dimensional properties of the human vestibulo-ocular reflex. in Proceedings of the XVth Bárány Society Meeting, eds Krejcova, H. and Jerabek, J., CNEXIM, Prague, pp. 44-47, 1993.

**1994**

**Zee**, D.S., Bedside evaluation of the dizzy patient. In: Dizziness and Balance Disorders,ed. I.K. Arenberg, Kugler, 175-182, 1994.

**Zee**, D.S., Vestibular adaptation. In: Vestibular Rehabilitation,ed. Susan J. Herdman, F.A. Davis, Philadelphia, 68-80, 1994.

Herdman, S., Sandusky, A., Hain, T.C, **Zee,** D.S., and Tusa, R.J., Characteristics of postural stability in patients with aminoglycoside toxicity. J. Vestib. Res., 4:71-80, 1994.

Tweed, D., Sievering, D., Misslisch, H., Fetter, M., **Zee**, D., and Koenig, E., Rotational kinematics of the human vestibuloocular reflex. I. Gain matrices, J. Neurophysiol . 72:2467-2479, 1994.

Fetter, M., **Zee,** D.S., Tweed, D., and Koenig, E., Head position dependent adjustment of the three-dimensional human vestibuloocular reflex. Acta Otolaryngol.,114:473-478, 1994.

Tiliket, C., Shelhamer, M., Roberts, D. and **Zee,** D.S., Short-term vestibulo-ocular reflex (VOR) adaptation in humans. I. Effect on the ocular motor velocity-to-position neural integrator. Exp. Brain Research, 100:316-327, 1994.

Shelhamer, M., Tiliket, C., Roberts, D. Kramer, P and **Zee,** D.S., Short-term vestibulo-ocular reflex (VOR) adaptation in humans. II. Error signals. Exp. Brain Research, 100:328-336, 1994.

Gauthier, G.M., Vercher, J-L., **Zee,** D.S., Changes in ocular alignment after sustained passive displacement of one eye. Vision Research, 34:2613-2627, 1994.

**Zee,** D.S., Adaptive control of eye movements: Clinical implications. The Canadian Journal of Neurological Sciences, 21:177-184, 1994.

Hain, T.C., Herdman, S.J., Holliday, M., **Zee**, D.S. and Byskosh, A.T., Localizing value of optokinetic afternystagmus. Annals of Otology, Laryngology and Rhinology,103:806-811, 1994.

Deuschl, G., Toro, C., Valls-Solé, J., Zeffiro, T., **Zee,** D.S., Hallett, M., Symptomatic and essential palatal tremor. 1. Clinical, physiological, and MRI analysis. Brain,117:775-788, 1994.

Ygge, J. and **Zee,** D.S.**,** Yoking of the eyes for vertical saccades. In Contemporary Ocular Motor and Vestibular Research: A Tribute to David A. Robinson eds. Fuchs, A.F., Brandt, T., Büttner, U., and **Zee,** D.S.**,** Thieme, Stuttgart, pp. 333-335, 1994.

Lewis, R. F., **Zee,** D.S. and Guthrie, B., Ocular proprioception contributes to disconjugate ocular motor adaptation. In Contemporary Ocular Motor and Vestibular Research: A Tribute to David A. Robinson, eds. Fuchs, A.F., Brandt, T., Büttner, U and **Zee,** D.S.**,** Thieme, Stuttgart, pp. 339-341, 1994.

**Zee,** D.S., Kramer, P, Shelhamer, M, and Tiliket, C. Adaptive control of the phase of the vestibuloocular reflex, in Contemporary Ocular Motor and Vestibular Research: A Tribute to David A. Robinson, eds. Fuchs, A.F., Brandt, T., Büttner, U., and **Zee,** D.S.**,** Thieme, Stuttgart, pp.52-59, 1994.

Fuchs, A.F., Brandt, T., Büttner, U., and **Zee,** D.S.**,** editors, Contemporary Ocular Motor and Vestibular Research: A Tribute to David A. Robinson, Thieme, Stuttgart, 1994.

Lewis, R. F., **Zee,** D.S. Gaymard, B. and Guthrie, B. Extraocular muscle proprioception functions in the control of ocular alignment and eye movement conjugacy. J. Neurophysiol. 72:1028-1031, 1994.

Gaymard, B., van der Drift, M. and **Zee,** D.S., Predictive saccadic eye tracking: Response to complex patterns of spatial and temporal stimuli. In Eye Movements in Reading, Ygge, J and Lennerstand, G, eds, Pergamon Press, pp 79-96, 1994.

**Zee,** D.S.**,** Disorders of adaptive control of eye movements: clinical implications. In International Workshop on Eye Movements, eds. M. Versino and D. Zambarbieri, Foundation IRCCS, Pavia, Italy, pp. 7-19, 1994.

**Zee,** D.S., Adaptation of the vestibuloocular reflex (VOR): Current concepts and diagnostic and therapeutic implications. in International Workshop on Eye Movements, eds. M. Versino and D. Zambarbieri, Foundation IRCCS, Pavia, Italy, pp. 184-200, 1994.

Yee, R.D., Spiegel, P.H., Yamad, T., Abel, L.A., Suzuki, D.A., and **Zee,** D.S., Voluntary saccadic oscillations, resembling ocular flutter and opsoclonus. J. Clinical Neuro-Ophthalmol . 14:95-101, 1994.

**Zee**, D.S., Bedside evaluation of the dizzy patient, in Advances in Otoneurology, eds. Pasali, D. and Nuti, D., Siena, 11-22, 1994.

**1995**

Frohman, E.M. and **Zee,** D.S., Ocular neuromyotonia (OMN): Clinical features, physiological mechanisms and response to therapy. Ann Neurol.37:620-626, 1995.

Knox, D.L, Green, Wm. R, Troncoso, J.C., Yardley, J.H., Hsu, J, and **Zee,** D.S., Cerebral ocular Whipple’s Disease: A 62-year odyssey from death to diagnosis. Neurology. 45:617-625, 1995.

**Zee**, D.S., Internuclear ophthalmoplegia. in: Neuroophthalmological Disorders: Diagnostic Workup and Management, R. J. Tusa and S. Newman, Decker, 489-506, 1995.

Straumann, D. and **Zee,** D.S.**,** Three-dimensional aspects of eye movements. Current Opinions in Neurology, 8:69-71, 1995.

Lewis, R.F, **Zee,** D.S., Repka, M.X., Guyton, D.L. and Miller, N.R., Regulation of static and dynamic ocular alignment in patients with trochlear nerve paresis. Vision Research, 35:3255-3264, 1995.

Kramer, P. D., Shelhamer, M. And **Zee,** D.S.**,** Short-term adaptation of the phase of the vestibuloocular reflex (VOR) in normal human subjects. Exp Brain Research, 106:318-326, 1995.

Ygge, J, and **Zee,** D.S., Control of vertical eye alignment in three dimensional space. Vision Research, 35:3169-3181, 1995.

Tsai, T-T, Lasker, A. and **Zee,** D.S., Visual attention in patients with Huntington's Disease: The effect of cueing on saccade latencies and manual reaction times. Neuropsychologia 33:1617-1626, 1995.

Takagi, M., Frohman, E. M., and **Zee,** D.S., Gap-overlap effects on latencies of saccades, vergence and combined vergence saccades. Vision Research, 35: 3373-3388, 1995.

Straumann, D., **Zee,** D.S.**,** Solomon D., Lasker, A.G., and Roberts D.C., Transient torsion and cyclovergence during and after saccades. Vision Research, 35:3321:3335, 1995.

**1996**

FitzGibbon, E., Calvert, P., **Zee,** D.S.**,** Dieterich, M., and Brandt, T., Torsional nystagmus during vertical smooth pursuit. J. of Neuroophthalmology, 16:79-90, 1996.

Tusa, R.J., Grant, M.P., Buettner, U., Herdman, S.H., and **Zee,** D.S., The contribution of the vertical semicircular canals to high-velocity horizontal vestibule-ocular reflex (VOR) in normal subjects and patients with unilateral vestibular nerve section. Acta Otolaryngol. 116:507-512, 1996.

Straumann, D., **Zee,** D.S.**,** Solomon, D., and Kramer, P.D., Validity of Listing's Law during fixation, saccades, smooth pursuit eye movements and blinks. Exp Brain Research, 112:135-146, 1996.

**Zee,** D.S. and Fletcher, W., Bedside evaluation of the dizzy patient. Eds. Baloh, R. and Halmagyi, M. H., in Disorders of the Vestibular System, Oxford Press. New York, pp 178-191, 1996.

Litvan, I. et al., Clinical research criteria for the diagnosis of progressive supranuclear palsy (Steele-Richarson-Olszewski syndrome). Report of the NINDS-SPSP international workshop. Neurology, 47:1-9, 1996.

**Zee,** D.S., Kramer, P., and Shelhamer, M., Short-term vestibuloocular (VOR) adaptation: contextual cues, adjustment of phase and the role of the neural integrator. In Collard, M., Jeannerod, M. and Christen, Y., Le Cortex Vestibulaire , Editions Irvinn, Paris, pp.65-72, 1996.

Frohman, E.M., Solomon, D., and **Zee,** D.S., Nuclear, supranuclear and internuclear eye movement abnormalities in multiple sclerosis. International Journal of MS, 2: 79-89, 1996.

Baloh, R.W., Fife, T.D., Furman, J.M. and **Zee,** D.S.**,** Neurootology Continuum, American Academy of Neurology, 1996.

Versino, M., Hurko, O. and **Zee,** D.S.**,** Disorders of binocular control of eye movements in patients with cerebellar dysfunction, Brain, 119:1933-1950, 1996.

**Zee,** D.S.**,** Considerations on the mechanisms of alternating skew deviation in patients with cerebellar lesions. J. Vestib. Research, 6:395-402, 1996.

**1997**

Solomon, D. Straumann, D. and **Zee,** D.S., Three dimensional eye movements during vertical axis rotation: Effects of visual suppression, orbital eye position and head position, in "Three-dimensional Kinematics of Eye, Head and Limb Movement", (M. Fetter, T. Haslwanter, H. Misslisch, D. Tweed, eds.) Harwood Academic Publishers, Amsterdam, 197-208, 1997.

Straumann, D, **Zee,** D.S., Solomon, D., Three-dimensional analysis of upward drift in cerebellar downbeat nystagmus, in "Three-dimensional Kinematics of Eye, Head and Limb Movement". (M. Fetter, T. Haslwanter, H. Misslisch, D. Tweed, eds.), Harwood Academic Publishers, Amsterdam, 243-250, 1997.

Lasker, A.G. and **Zee,** D.S.**,** Ocular motor abnormalities in Huntington's Disease (HD), Vision Research, 37:3639-3645, 1997.

Frohman, E.M., Solomon, D., and **Zee,** D.S., Vestibular dysfunction and nystagmus in multiple sclerosis. International Journal of MS, 3:13-26, 1997.

Carter, N. and **Zee,** D.S.**,** The anatomical localization of saccades using functional imaging studies and transcranial magnetic stimulation. Cur. Opin. Neurol., 10:10-17, 1997.

**Zee,** D.S.**,** Forward, "Three-dimensional Kinematics of Eye, Head and Limb Movement". (M. Fetter

T. Haslwanter, H. Misslisch, D. Tweed, eds.), Harwood Academic Publishers, Amsterdam, ix-x, 1997.

Gomez, C.M, Thompson, R.M., Gammack, J.T., Perlman, S.L., Dobyns, W.B., Truwit, C.L., **Zee,** D.S.**,** Clark, H.B., and Anderson, J.H., Spinocerebellar ataxia type 6: Gaze-evoked and vertical nystagmus, Purkinje cell degeneration, and variable age of onset. Ann. Neurol. 42:933-950, 1997.

**1998**

**Zee,** D. S., Supranuclear and internuclear disorders, in Miller, N. and Newman, N., Walsh and Hoyt’s Clinical Neuroophthalmology, Fifth Edition, Williams and Wilkins, pp. 1283-1381, 1998.

**Zee,** D.S.**,** Forward, "Dizziness, hearing loss and tinnitus", R. Baloh, F.A. Davis, Philadelphia, 1998.

Kramer, P.D., Shelhamer, M., Peng, G.C.Y. and **Zee,** D.S., Context-specific short-term adaptation of the phase of the vestibulo-ocular reflex. Exp Brain Res., 120:184-192, 1998.

Ming, X., Wang, M.M., **Zee,** D., Katz, R.M., and Freeman, J.M., Wernicke's encephalopathy in a child with prolonged vomiting, J Child Neurol. 13:187-189, 1998.

Kramer, P., Shelhamer, M. and **Zee,** D.S.**,** Short-term vestibuloocular (VOR) adaptation: influence of context, Otolaryngol, Head and Neck Surgery, 119: 60-64, 1998.

Kramer, P., Roberts, D., Shelhamer, M. and **Zee,** D.S., A versatile stereoscopic visual display for vestibular and oculomotor research, J Vestib. Res., 8:363-380, 1998.

Minor, L.B., Solomon, D., Zinreich, J.S. and **Zee,** D.S., Sound and/or pressure-induce vertigo due to bone dehiscence of the superior semicircular canal, Arch. Otolaryngol. Head Neck Surg. 124:249-258, 1998.

Kerrison, J.B., Koenekoop, R., Arnould, V.J., **Zee, D.,** and Maumenee, I.H., Clinical features of autosomal dominant congenital nystagmus linked to chromosome 6P12, Am. J. Ophthalmol. 125:64-70, 1998.

Takagi, M., **Zee,** D.S.**,** and Tamargo, R., Effects of lesions of the oculomotor vermis on eye movements in primate: saccades, J Neurophysiol., 80:1911-1931, 1998.

Hara, N., Steffen, H., Roberts, D.C., and **Zee**, D.S.**,** Effect of horizontal vergence on the motor and sensory components of vertical fusion, Invest Ophthalmol Vis Sci. 39:2268-2276, 1998.

Guyton, D.L., Cheeseman, E.W., Ellis, F.J., Straumann, D., **Zee,** D.S., Dissociated vertical deviation: an exaggerated normal eye movement used to damp cyclovertical latent nystagmus. Trans Amer. Ophthal. Soc., 96:389-429, 1998.

**Zee,** D.S.**,** Ocular motor control and the cerebellum: update. Jap. J. Neuroophthalmol .15:419-435, 1998.

Minor, L. and **Zee,** D.S., Evaluation of the dizzy patient. In Otolaryngology, Head and Neck Surgery, third edition, ed. C.W. Cummings et al. 2623-2671, 1998.

**1999**

Averbuch-Heller, L., Lewis, R.F., **Zee,** D.S., Disconjugate adaptation of saccades: contribution of binocular and monocular mechanisms, Vision Research,39:341-352, 1999.

Guyton, D.L., Cheeseman, E.W., Ellis, F.J., Straumann, D., **Zee,** D.S., Dissociated vertical deviation: etiology and mechanism. in Advances in Strabismology editor G. Lennerstrand, Aeolus Press,353-356, 1999.

**Zee**, D.S., Vestibular adaptation. In: Vestibular Rehabilitation, ed. Susan J. Herdman, F.A. Davis, Philadelphia, 77-90, 1999.

Leigh, R.J. and **Zee,** D.S.**,** The Neurology of Eye Movements, Third edition, Oxford University Press, New York, 1999.

Walker, M.F. and **Zee,** D.S. Hyperventilation and downbeat nystagmus in cerebellar patients. Neurology, 53:1576-1579, 1999.

Walker, M.F. and **Zee,** D.S. Directional abnormalities of vestibular and optokinetic responses in cerebellar disease. Ann. N.Y. Acad. Sci. 871:205-220, 1999.

Hegemann, S., Shelhamer, M.J., **Zee,** D.S., Phase adaptation of the linear vestibulo-ocular reflex. Ann. N.Y. Acad. Sci., 871:414-416, 1999.

Minor, L., Haslwanter, T., Straumann, D., **Zee,** D.S., Hyperventilation-induced nystagmus in patients with vestibular schwannoma. Neurology, 53:2158-2168, 1999.

Lewis, R.F., **Zee,** D.S., Goldstein, H.P., Guthrie, B.L., Proprioceptive and retinal afference modify post-saccadic drift. J. Neurophysiology, 82:551-563, 1999.

W. Heide, E. Koenig, P. Trillenberg, D. Kömpf, D.S. **Zee**, Electrooculography: technical standards and applications, in, G. Deuschl and A. Eisen (eds.) Recommendations for the Practice of Clinical Neurophysiology (RCN). Second Edition 1999. Electroencephalography and Clinical Neurophysiology Supplement Series. 52: 223-240, 1999.

Frohman, E., **Zee,** DS, Supranuclear eye movement disorders. Oxford Textbook of Ophthalmology, Editors: Easty, D, Sparrow, J., chapter 2.12.9, 1999.

Walker, M.F. and **Zee,** D.S., Eye movement recordings in the evaluation of ophthalmologic and neurologic disorders. Curr. Opin. Ophthalmol., 10: 401-404, 1999.

**Zee,** D.S., Book Review, Vertigo: Its Multisensory Syndromes. in TINS, 23:40, 1999.

**2000**

Takagi, M., **Zee,** D.S., Tamargo, R., Effects of lesions of the oculomotor cerebellar vermis on eye movements in primate: Smooth pursuit. J. Neurophysiol., 83:2047-2062, 2000.

Walker, M.F. and **Zee,** D.S., Bedside vestibular examination. Otolaryngol Clinic N. America, 33:495-506, 2000.

Taylor, M. J., Roberts, D.C. and **Zee,** D.S.**,** Effect of sustained cyclovergence on eye alignment: Rapid torsional phoria adaptation, Invest Ophthalmol Vis Sci . 41:1076-1083, 2000.

Straumann, D., **Zee,** D.S., Solomon, D., Three-dimensional kinematics of ocular drift in humans with cerebellar atrophy. J. Neurophysiol., 83:1125-1140, 2000.

Steffen, H., Walker, M.F. and **Zee,** D.S., Rotation of Listing's plane with convergence: independence from eye position. Invest Ophthalmol Vis Sci. 41:715-721, 2000.

Lee, C., **Zee,** D.S.**,** and Straumann, D., Saccades from torsional offset positions back to Listing’s plane. J. Neurophysiol. 83:3241-3253, 2000.

Steffen, H., Hara, N., Roberts, D.C. and **Zee,** D.S.**,** Motor and sensory responses in fusion of vertical disparities in different convergence places. Opthalmologie, 97:16-21, 2000.

Takagi, M, Abe, H, Hasegawa, S, Usui, T, Hasebe, H, Miki, **Zee**, D.S., Context-specific adaptation of pursuit initiation in humans. Invest Ophthalmol Vis Sci 41:3763-3769, 2000.

Goldberg, M.D., Landa, R., Lasker, A., Cooper, L., and **Zee,** D.S.**,** Evidence of normal cerebellar control of the vestibulo-ocular reflex (VOR) in children with high functioning autism. J. Autism Dev Disorders,30:519-524, 2000.

Fife, T.D., Tusa, R.J., Furman, J.M., **Zee,** D.S., Baloh, R.W., Hain, T., Goebel, J., Frohman, E., Demer, J., Eviatar, L., Assessment: Vestibular testing techniques in adults and children. Neurology,55:1431-1441, 2000.

Walker, M., **Zee,** D.S., Cerebellar control of gaze, in Neuroophthalmology at the Beginning of the Millenium. Ed. James Sharpe, Medimond Publishing, New Jersey, pp 71-82, 2000.

Walker, M.F. and **Zee,** D.S.**,** Approach to the patient with dizziness and vertigo. Kelly’s Text Book of Medicine. pp 2844-2866, 2000.

Hegemann, S., Shelhamer, M., Kramer, P., **Zee,** D.S., Adaptation of the phase of the human linear vestibulo-ocular reflex (LVOR) and effects on the ocular motor neural integrator**.** J. Vestib. Res. 10:239-247, 2000.

Shelhamer, M., Roberts, D.C., **Zee,** D.S., Dynamics of the human linear vestibulo-ocular reflex (LVOR) at medium frequency and modification by short-term training. J. Vestib. Res., 10:271-282, 2000.

**2001**

Mejico, L., **Zee,** D.S., New insights into the pathogenesis and treatment of Ménières disease, Neurology Network, 4:1-11, 2001.

Takagi, M., Oyamada, H., Abe, H., **Zee,** D.S., Hasebe, H., Miki, A., Usui, T., Hasegawa, S., Bando, T., Adaptive changes in dynamic properties of human disparity-induced vergence. Invest. Ophthalmol. Vis. Sci 42:1479-1486, 2001.

Bergamin, O., Straumann, D., **Zee,** D.S., Roberts, D.C., Landau, K., Lasker, A.G., Three-dimensional Hess screen test with binocular dual search coils in a three-field magnetic system. Invest Ophthalmol Vis Sci., 42:660-667, 2001.

Ramat, S., **Zee,** D.S., Minor, L.B., Translational vestibuloocular reflex evoked by a 'head heave' stimulus. Ann. NY Acad., Sci., 942:95-113, 2001.

Takagi, M, Trillenberg, P., **Zee,** D.S., Adaptive control of eye movements in humans: control of smooth pursuit, vergence and eye torsion. Vision Research, 41:3329-3342, 2001.

Mostofsky, S.H., Lasker, A.G., Cutting, L.E., Denckla, M.B., **Zee,** D.S., Oculomotor abnormalities in Attention Deficit Hyperactivity Disorder: a preliminary study. Neurology,57:423-430, 2001.

Guyton, D., **Zee,** D.S.**,** Further evidence that DVD serves to damp or block latent nystagmus, Transactions 27th Meeting European Strabismological Association,pp 101-104, 2001.

# Mostofsky S.H., Lasker A.G., Singer H.S., Denckla M.B., and Zee D.S., Oculomotor abnormalities in children with Tourette syndrome with and without ADHD. Journal American Academy Child Adolescent Psychiatry,40:1464-1472, 2001.

# Lewis, R.L., Zee, D.S., Hayman, M.R, Tamargo, R.J., Oculomotor function in rhesus monkey after deafferentation of the extraocular muscles, Exp Brain Research, 141:349-358, 2001.

**2002**

Steffen, H., Walker, M., **Zee,** D.S., Changes in Listing's plane following sustained vertical fusion. Invest Ophthalmol Vis. Sci 43:668-672, 2002.

Mudgil, A.V., Walker, M., Steffen, H., Guyton, D.L., **Zee,** D.S., Motor mechanisms of vertical fusion in individuals with superior oblique paresis. JAAPOS, 6:145-153, 2002.

Trillenberg, P., **Zee,** D.S.**,** Shelhamer, M.J., On the distribution of fast phases in OKN and VOR, Biological Cybernetics, 87:67-78, 2002.

**Zee,** D.S., Walker, M.F., Ramat, S., The cerebellar contribution to eye movements based upon lesions: binocular, three-axis control and the translational vestibulo-ocular reflex. Ann NY Acad. Sci. 956:178-189, 2002.

Walker, M.F., **Zee,** D.S.**,** Rectified cross-axis adaptation of the vestibulo-ocular reflex in rhesus monkey. Ann NY Acad Sci. 956:543-545, 2002.

Rosenberg M., **Zee,** D.S.**,** Unilateral rebound nystagmus: one manifestation of two different pathologic processes. Ann NY Acad Sci., 956: 585-587, 2002.

Ramat, S. **Zee,** D.S., TVOR responses to abrupt interaural accelerations in normal humans. Ann NY Acad Sci., 956:551-554, 2002.

Kramer, P. Frohman, E., Nuti, D., **Zee,** D.S.**,** The effect of horizontal head position (yaw axis) and step velocity on the vestibulo-ocular reflex. Ann NY Acad Sci.,956:530-532, 2002.

Kuniyoshi, S.M., Riley, D.E., **Zee,** D.S.**,** Reich, S.G., Leigh, R.J., Evaluation of clinical signs to differentiate PSP from Parkinson’s disease. Ann N.Y. Acad. Sci. 956: 484-486, 2002.

Goldberg, M.C., Lasker, A.G., **Zee,** D. S., Garth, E., Tien, A., Landa, R.J., Deficits in the initiation of eye movements in the absence of a visual target in adolescents with high functioning autism. Neuropsychologia 40:2039-2049, 2002.

Miller, N.R., Biousse, V., Wang, T., Patel, S., Newman, N.J., **Zee,** D.S., Isolated acquired unilateral horizontal gaze paresis from a putative lesion of the abducens nucleus. J. Neuroophthalmol . 22:204-207, 2002.

Ramulu, R., Moghekar, A., Chaudhry, V., **Zee,** D.S.**,** Reich, S.G., Wernicke’s encephalopathy (Neuroimage), Neurology 59:846, 2002.

**Zee**, D.S., and Leigh, R.J., Ocular motor control -- normal and abnormal. In: Diseases of the Nervous System, eds., Asbury, A., McKhann, G.M. and McDonald, W.I, Mcarthur, J., Cambridge University Press 634-657, 2002.

Walker, M.F. and **Zee**, D.S., Three-axis eye movement abnormalities with cerebellar lesions. Ann NY Acad Sci, 978:547, 2002.

Walker, M.F., Steffen, H., **Zee,** D.S.**,** Three-axis approaches to ocular motor control: a role for the cerebellum, in "Levels of Perception", eds. L. Harris, M. Jenkin. Springer-Verlag, 399-413. 2002.

**2003**

Trillenberg, P., Shelhamer, M., Roberts, D.C., **Zee,** D.S., Cross-axis adaptation of torsional components in the yaw-axis VOR, Exp Brain Res. 148:158-165, 2003.

Takagi, M., Tamargo, R., **Zee,** D.S.**,** Effects of lesions of the cerebellar oculomotor vermis on eye movements in primate: binocular control. in, Neural Control of Space Coding and Action Production eds. Prablanc, C., Pélisson, D., Rossetti, Y., Progress in Brain Research, 142:19-33, 2003.

Lasker, A.G., Denckla, M.B., **Zee,** D.S.**,** Ocular motor behavior of children with Neurofibromatosis 1 (NF-1), J Child Neurol 18:348-355, 2003.

Seemungal, B., Faldon, M., **Zee,** D.S., Bronstein, A**.,** Influence of target size on vertical gaze palsy in a patient with progressive supranuclear palsy, Movement Disorders,18: 818-822, 2003.

Moo, L.R., Slotnick, S.D. Tesoro, M.A. **Zee,** D.S.**,** Hart, J Jr. Interlocking-Finger Test: A bedside screen for parietal lobe dysfunction. J Neuro Neurosurg Psych. 74:530-2, 2003.

Lewis, R.F., Clendaniel, R.A., **Zee,** D.S., Vergence-dependent adaptation of the vestibulo-ocular reflex, Exp Brain Res,152:335-340, 2003.

Solomon, D., **Zee,** D.S.**,** Straumann, D., Torsional and horizontal vestibuloocular reflex adaptation: three dimensional eye movement analysis, Exp Brain Res 152:150-5, 2003.

Ramat, S. and **Zee,** D.S., Ocular motor responses to abrupt interaural head translation in normal humans, J Neurophysiol 90:887-902, 2003.

Lustig, L.H., Niparko, J.K., editors, Minor, L.B, **Zee,** D.S. Associate editors, Clinical Neurootology, Martin Dunitz, London, 2003.

Minor, L.B. and **Zee,** D.S.**,** Clinical evaluation of the patient with dizziness, in Lustig, L.H., Niparko, J.K., editors, Minor, L.B, **Zee,** D.S., associate editors, Clinical Neurootology, Martin Dunitz, London, 81-110, 2003.

Minor, L.B., Hullar, T.E., **Zee,** D.S.**,** Anatomy and physiology of the vestibular system. In Lustig, L.H., Niparko, J.K., editors, Minor, L.B, **Zee,** D.S., associate editors, Clinical Neurootology Martin Dunitz, London, 2003.

Eggers, S.D.Z. and **Zee,** D.S., Evaluating the dizzy patient: Bedside examination and laboratory assessment of the vestibular system. Sem. Neurology,23:47-58, 2003.

Straumann, D., Steffen, H., Landau, K., Bergamin, R., Mudgil, A.V., Walker, M.F., Guyton, D.L., **Zee,** D.S., Primary position and Listing's law in congenital and acquired trochlear nerve palsy, Invest Ophthalmol Vis Sci,,44:282-292, 2003.

**Zee,** D.S. and Walker, M.F., Cerebellar control of eye movements. In: The Visual Neurosciences. L.M. Chalupa and J.S. Werner (Eds.). Cambridge, MA: MIT Press, 1485-1498, 2003.

Frohman T.C., Frohman E.M., O'Suilleabhain P., Salter A., Dewey R.B. Jr., Hogan N., Galetta S., Lee A.G., Straumann D., Noseworthy J., **Zee** D., Corbett J., Corboy J., Rivera V.M., Kramer P.D., Accuracy of clinical detection of INO in MS: Corroboration with quantitative infrared oculography. Neurology,61:848-850, 2003.

Eggers, S.D.Z., **Zee,** D.S., Evaluating the dizzy patients: Bedside examination. Revista de Medicina, 47:11-20, 2003.

**Zee,** DS**,** Adaptive control of pursuit eye movements in humans. Strabismus,11:243-245, 2003.

Eggers, S.D.Z., de Pennington, N., Walker, M.F., Shelhamer, M., **Zee,** D.S., Short-term adaptation of the VOR: Non-retinal slip error signals and saccade substitution. Ann NY Acad Sci., 1004:94-110, 2003.

Shelhamer, M., Ramat, S., **Zee,** D.S.**,** Context-specific adaptation and its significance for neurovestibular problems of space flight. J Vest Res. 13:345-362, 2003.

**2004**

Peng, G. C-Y., **Zee**, **D.S**., Minor, L.B., Phase-plane analysis of gaze stabilization to high acceleration head thrusts – a continuum across normal subjects and patients with loss of vestibular function. J Neurophysiol. 91:1763-1781, 2004.

Bergamin, O., Ramat, S., Straumann, D., **Zee,** D.S.**,** Influence of orientation of exiting wire of search coil annulus on torsion following saccades. Invest Ophthalmol Vis Sci.,45:131-7, 2004.

Walker, M.F., Shelhamer, M., **Zee,** D.S., Eye position dependency of torsional eye velocity during interaural translation, horizontal pursuit, and yaw axis rotation in humans. Vision Research. 44:613-620, 2004.

**Zee,** D.S., Editorial review – Neuro-ophthalmology and Neuro-otology, Curr Opin Neurol. 17:1-2, 2004.

McKenna, G.J., Peng, G. C-Y., **Zee,** D.S.**,** Neck vibration alters visually perceived roll in normals, JARO, 5:25-31, 2004.

Tanimoto, N., Takagi, T., Bando, T., Abe, H., Hasegawa, S., Usui, T., Miki, A., **Zee,** DS, Central and peripheral visual interactions in disparity-induced vergence eye movements: I. Spatial interaction. Invest Ophthalmol Vis Sci., 45:1132-1138, 2004.

**Zee,** D.S. and Lasker, A., Antisaccades: probing cognitive flexibility using eye movements. Neurology, 63:1554, 2004.

**Zee,** DS and Newman-Toker- D., Supranuclear and internuclear disorders, in Walsh and Hoyt’s Neuroophthalmology, Miller, N and Newman, N. eds. Lippincott William Wilkins, chapter 19, pp 907-969, vol. 1, 2004.

**2005**

Ramat, S., Leigh, R.J., **Zee,** D.S.**,** Optican, L.M., Saccadic oscillations may be caused by coupling of brainstem excitatory and inhibitory burst neurons, Exp Brain Res , 160: 89-106, 2005.

Solomon, D., Winkelman, A.D., **Zee,** D.S.**,** Gray, L., Büttner-Ennever, J. Niemann-Pick Type C disease in two affected sisters: ocular motor recordings and brainstem neuropathology. Ann NY Acad Sci, 1039:436-435, 2005.

Ying, SH, Choi, SI , Lee, M , Perlman, SL , Baloh, RW , Toga, AW, **Zee** DS, Relative atrophy of the flocculus and ocular motor dysfunction in SCA2 and SCA6, Ann NY Acad Sci. 1039, 430-435, 2005.

Ramat, S. and **Zee,** D.S., Binocular coordination in fore/aft motion. Ann NY Acad Sci. 1039:36-53, 2005.

Nuti, D, Mandalà, M, Broman AT, and **Zee,** DS**,** Acute vestibular neuritis: Prognosis based upon bedside clinical tests (thrusts and heaves). Ann NY Acad Sci. 1039:359-367, 2005.

Lasker; A.G. Isotalo, E.H., **Zee,** D.S. Predictive saccades to a regularly alternating target in cerebellar patients. Ann NY Acad Sci. 1039, 544-547, 2005.

Peng, GCY, Minor, L, **Zee,** D.S.**,** Gaze position corrective eye movements in normal subjects and in patients with vestibular deficits. Ann NY Acad Sci. 1039:337-348, 2005.

Walker, M., and **Zee,** D.S., Asymmetry of the pitch vestibulo-ocular reflex in patients with cerebellar disease. Ann NY Acad Sci. 1039: 349-358, 2005.

Frohman, EM, Frohman, TC, **Zee,** DS**,** McColl, R., Galetta, S. The neuroophthalmology of multiple sclerosis, Lancet Neurology, 4: 111-121, 2005.

Ramat, S., Straumann, D. and **Zee,** D.S., The interaural translational VOR: suppression, enhancement and cognitive control, J Neurophysiol. 94, 2391-2402, 2005.

Isotalo, E.H., **Zee,** D.S., Lasker, A. G., Cognitive influences on predictive saccade tracking, Exp Brain Res.

165:461-469, 2005.

Hullar, T.E., Minor, L.B., **Zee,** D.S., Evaluation of the patient with dizziness. Chapter 140, 3160-3199., in Cummings CC., et al. Otolaryngology Head & Neck Surgery, Vol 3, Elsevier Mosby, Philadelphia, 2005.

Eggers, S.D.Z. and **Zee,** D.S., Central vestibular disorders. Chapter 143, 3254-3290, in Cummings CC., et al. Otolaryngology Head & Neck Surgery, Vol 3, Elsevier Mosby, Philadelphia, 2005.

Leigh, R.J. and **Zee,** D.S.**,** Role of ocular motor assessment in diagnosis and research, Chapter 15, pp. 233:252 in Atypical Parkinsonian Disorders, ed. Litvan, I., Human Press, 2005

Walker, M., and **Zee,** D.S.**,** Cerebellar disease alters the axis of the high-acceleration vestibulo-ocular reflex, J. Neurophysiol.,94: 3417-3529, 2005.

**2006**

Migliaccio, A.A., Schubert, M.C., Clendaniel, R.A., Carey, J.P., Della Santina, C.C., Minor, L.P., **Zee,** D.S., Axis of eye rotation changes with head-pitch orientation during head impulses about Earth-vertical, JARO, 7:140-150, 2006.

Leigh, R.J. and **Zee,** D.S.**,** The Neurology of Eye Movements, Fourth edition, Oxford University Press, New York, 2006.

Migliaccio, A.A., Della Santina, C.C., Carey, J.P., Minor, L.P., **Zee,** D.S. The effect of binocular eye position and head rotation plane on the human torsional vestibuloocular reflex**,** Vision Res, 46:2475-86, 2006.

Ying, S., Choi, S., Perlman, S., Baloh, R., **Zee,** D.S, Toga, A., Pontine and cerebellar atrophy correlate with clinical disability in SCA2, Neurology,66:424-426, 2006.

Ying, S. and **Zee,** D.S., Phoria adaptation after sustained symmetrical convergence: influence of saccades, Exp Brain Res, 171: 297-305, 2006.

Tian, J., **Zee,** D.S.**,** Walker, M., Eye-position dependence of torsional velocity during step-ramp pursuit and transient yaw rotation in humans, Exp Brain Res, 171:225-230, 2006.

**Zee,** D.S., Pathophysiology of vestibular symptoms and signs: The clinical examination. Continuum: Lifelong Learning in Neurology. 12:13-32, 2006.

**2007**

Lasker, A.G., Mazzocco, M., **Zee,** D.S. Ocular motor indicators of executive dysfunction in Fragile X and Turner syndromes. Brain and Cognition, 63:203-220, 2007.

Ramat, S, Leigh, R.J., **Zee,** D.S.**,** Optican, L.M., What human eye movement disorders tell us about the neural control of saccades, Brain, 130:10-35, 2007.

Tarnutzer, A., Ramat, S., Straumann, D., **Zee,** D.S., Pursuit responses to target steps during ongoing tracking. J Neurophysiol., 97:1266-1279, 2007.

Tian, J., **Zee,** D.S.**,** Walker, M.F, Rotational and translational optokinetic nystagmus have different kinematics, Vision Res, 47: 1003-1010, 2007.

**Zee**, D.S., Vestibular adaptation. In: Vestibular Rehabilitation, 3^rd^ Edition, ed. Susan J. Herdman, F.A. Davis, Philadelphia, 19-28, 2007.

Shan, X, Tian, J, Ying, H, Quaia, C, Optican, L, Walker, M, Tamargo, R., **Zee,** D.S., Acute superior oblique palsy in monkeys: I. Changes in static eye alignment, Invest Ophthalmol Vis Sci, 48:2602-2611, 2007.

Shan, X, Tian, J, Ying, H, Quaia, C, Optican, L, Walker, M, Tamargo, R., **Zee,** D.S., Acute superior oblique palsy in monkeys: II. Changes in dynamic properties during vertical saccades, Invest Ophthalmol Vis Sci, 48:2612-2620, 2007.

Tian, J, Shan, X, **Zee,** D.S., Ying, H, Quaia, C, Optican, L, Tamargo, R., Walker, M, Acute superior oblique palsy in monkeys: III. Changes in Listing’s law behavior, Invest Ophthalmol Vis Sci, 48:2621-2625, 2007.

Walker, M., Tian, J., **Zee,** D.S., Kinematics of the rotational vestibulo-ocular reflex: role of the cerebellum and implications for neural control of Listing’s law, J. Neurophysiol., 98:295-302, 2007.

Ying, H.S., Darbandi, B., Shan, X., Barker, P., Miller, N.R., **Zee,** D.S., Quantitative eye movement recordings in a patient with bilateral superior oblique palsy after Harada-Ito procedure, Strabismus, 15:137-147, 2007.

Newman-Toker, D.E., Cannon, L.M., Stofferahn, M.E., Rothman, R.E., Hsieh, Y-H, **Zee** D.S., Patient reports of symptom quality are imprecise: A cross-sectional study conducted in an acute care setting, Mayo Clinic Proceed., 82: 1329-1340, 2007.

Shaikh, A.G., Miura, K., Optican, L.M., Ramat, S., Leigh, R.J., **Zee,** D.S., A new familial disease of saccadic oscillations and limb tremor provides clues to mechanisms of common tremor disorders. Brain, 130:3020-3031, 2007.

**2008**

Shaikh, A.G., Jinnah, H.A., Tripp, R.M., Optican, L.M., Ramat, S., Lenz, F.A., **Zee,** D.S., Irregularity distinguishes limb tremor in cervical dystonia from essential tremor. J Neurol Neurosurg Psychiatry, 79:187-189, 2008.

Chen-Harris, H., Joiner, W.M., Ethier, V., **Zee,** D.S., Shadmehr, R., Adaptive control of saccades via internal feedback. J. Neuroscience, 28:2804-13, 2008.

Shan, X, Tian, J, Ying, H, Walker, M,, Guyton, D, Quaia, C, Optican, L, Tamargo, R., **Zee,** D.S.**,** The effect of acute superior oblique palsy on torsional optokinetic nystagmus in monkeys. Invest Ophthalmol Vis Sci, 49:1421-1428, 2008.

Frohman, T., Galetta, S., Fox, R., Solomon, D., Straumann, D., Filippi, M., **Zee,** D., Frohman, E. Pearls and Oysters: The medial longitudinal fasciculus (MLF) in ocular motor physiology, Neurology, 70:57-67, 2008.

Mandalà , M., Nuti, D., Broman, A., **Zee,** D.S.. Can a careful bedside examination be effective in the assessment, diagnosis and prognosis of vestibular neuritis? Arch. Otolaryngol. Head and Neck Surgery, 134:164-69, 2008.

Ethier, V., **Zee,** D.S., Shadmehr, R., Spontaneous recovery of motor memory during saccade adaptation, J Neurophysiology, 99:2577-83, 2008.

Solomon, D., Ramat, S., Tomsak, R., Reich, S., Shin, R., **Zee,** D.S.**,** Leigh, R.J., Saccadic palsy following cardiac surgery: Characteristics and pathogenesis. Ann Neurol, 63:355-365, 2008.

Steffen, H., Straumann, D.S., Walker, M.F., Miller, N.R., Guyton, D.L., Repka, M.X., **Zee,** D.S., Torsion in patients with superior oblique palsies: dynamic torsion during saccades and changes in Listing’s plane, Graefe's Arch Clinical Exp Ophthalmol, 246:771-778, 2008.

Mills, D. Frohman, T.C., Davis, S., Salter, A., McClure, S., Beatty, I., Shah, A., Galetta, S., Eggenberger, E., **Zee,** D.S.**,** Frohman, E.M., Break in binocular fusion during head turning in MS patients with INO. Neurology, 71: 458-460, 2008.

Liao, K., Hong, S., **Zee,** DS**,** Optican, LM, Leigh, RJ,Using wavelet analysis to evaluate effects of eye and head movements on ocular oscillations. in Advances in Understanding Mechanisms and Treatment of Infantile forms of Nystagmus, eds. Leigh, RJ and Devereaux, MW. Oxford University Press, pp184-188, 2008.

Walker, M.F., Tian, J., Shan, X., Tamargo, R.J., Ying, H., and **Zee,** D.S., Effects of cerebellar lesions in monkeys on gaze stability. in Advances in Understanding Mechanisms and Treatment of Infantile forms of Nystagmus, eds. Leigh, RJ and Devereaux, MW. Oxford University Press, pp 55-60, 2008

Walker, M., Tian, J., Shan, X., Tamargo, R., Ying, H., **Zee,** D.S., Lesions of the cerebellar nodulus and uvula impair downward pursuit, J Neurophysiology, 100,1813-1823, 2008.

Quaia, C., Shan, X., Tian, J., Ying, H., Optican, L.M., Walker, M., Tamargo, R., **Zee,** D.S., Acute superior oblique palsy in the monkey: effects of viewing conditions on ocular alignment and modeling of the ocular motor plant. Progress Brain Res, 171, 47-52, 2008.

Sinha, N, Zaher, N, Shaikh, A, Lasker, A, **Zee,** DS, Tarnutzer, AA, Perception of self-motion during and after passive rotation of the body around an earth-vertical axis, Progress Brain Res, 171,227-281, 2008

Walker, M, Tian, J, Shan, X, Tamargo, R, Ying, H, **Zee,** DS, Lesions of the cerebellar nodulus and uvula in monkeys: effect on otolith-ocular reflexes. Progress Brain Res, 171: 167-172, 2008.

Ramat, S., Leigh, RJ, **Zee,** DS**,** Shaikh, A, Optican, LM., Applying saccade models to account for oscillations, Progress Brain Res, 171, 123-130, 2008.

Bertolini, G., Bockisch, CJ, Straumann, D, **Zee,** DS**,** Ramat, S., Do humans show velocity storage in the vertical rVOR, Progress Brain Res, 171, 207-210, 2008.

Bertolini, G., Bockisch, CJ, Straumann, D, **Zee,** DS**,** Ramat, S., Examining the time constant of the pitch rVOR by separation of otoliths and semicircular canals contributions. Conf Proc IEEE Eng Med Biol Soc 1:1060-63, 2008.

Liao, K, Hong, S, **Zee,** DS, Optican, LM and Leigh, RJ. [Impulsive head rotation resets oculopalatal tremor: examination of a model](http://www.sciencedirect.com/science?_ob=GatewayURL&_method=citationSearch&_urlVersion=4&_origin=SDVIALERTHTML&_version=1&_uoikey=B7CV6-4T7WBSW-18&md5=9d8fb91ebac5d192ec249ca63bb7ef7f), Progress Brain Res, 171, 227-234, 2008.

Hong, S, Leigh, RJ, **Zee,** DS and Optican, LM, [Inferior olive hypertrophy and cerebellar learning are both needed to explain ocular oscillations in oculopalatal tremor](http://www.sciencedirect.com/science?_ob=GatewayURL&_method=citationSearch&_urlVersion=4&_origin=SDVIALERTHTML&_version=1&_uoikey=B7CV6-4T7WBSW-17&md5=a14f3025614675747f3c1208599408f5), Progress Brain Res, 171, 219-226, 2008.

Olasagasti, I, Bockisch, CJ, **Zee,** DS and Straumann, D. [Cyclovergence evoked by up–down acceleration along longitudinal axis in humans](http://www.sciencedirect.com/science?_ob=GatewayURL&_method=citationSearch&_urlVersion=4&_origin=SDVIALERTHTML&_version=1&_uoikey=B7CV6-4T7WBSW-1S&md5=5c6d2bd495ddfdcb2b41bd2848988d38), Progress Brain Res, 171, 319-322, 2008.

Solomon, D, Ramat, S., Leigh, RJ, **Zee,** DS. [A quick look at slow saccades after cardiac surgery: where is the lesion?](http://www.sciencedirect.com/science?_ob=GatewayURL&_method=citationSearch&_urlVersion=4&_origin=SDVIALERTHTML&_version=1&_uoikey=B7CV6-4T7WBSW-35&md5=4698400b3757f043510b15017b5a211f) Progress Brain Res, 171, 587-590, 2008.

Tian, J, Shan, X, Ying, H, Tamargo, R., Walker, M, **Zee,** D.S., The effect of acute superior oblique palsy on vertical pursuit in monkeys, Invest Ophthalmol Vis Sci, 49, 3927-3932, 2008.

Ethier, V, **Zee,** DS, Shadmehr, R, Changes in control of saccades during gain adaptation, J. Neurosci.,28:13929-13937, 2008.

Shaikh, AG, Miura, K, Optican, LM, Ramat, S., Tripp, RM., **Zee,** DS**,** Hypothetical membrane mechanisms in essential tremor, J. Translational Medicine, 6:68, 2008.

Newman-Toker, D, FJ, Stanton, VA, **Zee,** DS, Calkins, H, Robinson, KA, How often is dizziness from cardiovascular disease true vertigo? A systematic review. J Gen Internal Medicine, 23: 2087-94, 2008.

Shaikh, A.G., Ramat, S., Optican, L.M., Miura, K., Leigh, R.J., **Zee,** D.S., Saccadic burst cell membrane dysfunction is responsible for saccadic oscillations, J Neurophthalmol, 28: 329-336, 2008.

**Zee,** DS and Shaikh, AG, Think membranes and ion channels, Curr Opin Neurol, 21:1-2, 2008.

**2009**

**Zee,** DS, and Walker, M, Cerebellum and oculomotor control, in Squire, LR (ed), Encyclopedia of Neuroscience, third edition, vol. 2, pp 729-736, Elsevier, Press, 2009.

Tian, J, Ethier, V., Shadmehr, R., Fujita, M., **Zee,** D.S., Some perspectives on saccade adaptation, Ann NY Acad Sci, 1164: 166-172, 2009.

Ishii L, Carey J, Byrne P, **Zee** D. S., Ishii M, Measuring attentional bias to peripheral facial deformities, The Laryngoscope,119::459-465, 2009.

Shaikh, AG, Marti, S, Tarnutzer, AA, Palla, A, Crawford, TO, Straumann, D, Taylor, AM, **Zee,** DS, Gaze fixation deficits and their implication in ataxia-telangiectasia, J Neurol Neurosurg Psychiatry 80:855-864, 2009.

Newman-Toker, D, Sharma, P, Chowdhury, M, Clemmons, T., **Zee,** DS, Della Santina, CC. Penlight –Cover Test A New Bedside Method to Unmask Nystagmus, J Neurol Neurosurg Psychiatry, 80:900-903, 2009.

Mahoney,E M. Mostovsky, SH, Lasker, AG, **Zee,** D, Denkla, MB. Oculomotor Anomalies in Attention-Deficit/Hyperactivity Disorder: Evidence for Deficits in Response Preparation and Inhibition. J Am. Acad. Child Adol. Psych. 48:749-756, 2009.

Hong, C, Harris, JC, Pearlson, G, Kim, JS, Calhoun, V, Fallon, JH, Golay, X, Gillen, JS, Simmonds, DJ, van Zihl, P, **Zee,** DS, Pekar, J fMRI evidence for multisensory recruitment associated with rapid eye movements during sleep. Human Brain Mapping, 30:1705-22, 2009.

Xu-Wilson, M, **Zee,** DS, Shadmehr, R. The intrinsic value of visual information affects saccade velocities, Exp Brain Research, 196:475-481, 2009.

Walker, M, Tian, J, Shan, X Tamargo, R, Ying, H, **Zee,** DS, Enhancement of the bias component of downbeat nystagmus after lesions of the nodulus and uvula. Ann NY Acad Sci, 1164: 482-485, 2009.

Ying, SH, Landman, BA, Chowdhury, S, Sinofsky, AH, Gambini, A, Mori, S, **Zee,** DS, Prince, JL, Orthogonal diffusion-weighted MRI measures distinguish region-specific degeneration in cerebellar ataxia subtypes, J Neurology, 256: 1939-42, 2009.

[Xu-Wilson](Javascript:%20view_member('Haiyin',%20'Chen');), M, [Chen-Harris](Javascript:%20view_member('Wilsaan',%20'Joiner');), H, [**Zee,**](Javascript:%20view_member('David',%20'Zee');) DS, Shadmehr, R, Cerebellar contributions to adaptive control of saccades in humans, J Neuroscience, 29: 12930-39, 2009.

**2010**

Eggers, Scott DZ and **Zee,** DS, editors, Vertigo and Imbalance: Clinical Neurophysiology of the Vestibular System, volume 9, Handbook of Clinical Neurophysiology, Amsterdam, Elsevier, 2010.

Shaikh AG, Hong S, Liao K, Tian J, Solomon D, **Zee** DS, Leigh RJ, Optican LM, Oculopalatal tremor explained by model with inferior olivary hypertrophy and cerebellar plasticity, Brain, 133: 923-940, 2010.

Shaikh, AG, Reich, S., **Zee,** DS, Pseudonystagmus, Nature Reviews Neurology, 6:519-23, 2010.

Helminski, J, **Zee,** DS, Hain, TC, Effectiveness of particle repositioning maneuvers in the treatment of benign paroxysmal positional vertigo: A systematic review. Physical Therapy, 90:663-78, 2010.

Schubert, M. **Zee,** DS, Saccades and vestibular ocular motor adaptation, Restor Neurol Neurosci, 28:9-18, 2010.

Shaikh, AG, Hain, TC, **Zee,** DS, Oculomotor disorders in adult-onset Still’s disease, J Neurology, 257:136-138, 2010.

Demer, J, Poukens, V, Ying, H, Shan, X, Tian, J, **Zee** DS, Effects of intracranial trochlear neurectomy on the structure of the primate superior oblique muscle, Invest Ophthalmol Vis Sci, 51:3485-93, 2010.

Du, A, Bennett, L. **Zee,** DS, Mori, S, Prince, JL, Ying, SH, Diffusion tensor imaging reveals disease-specific deep cerebellar nucleus changes in cerebellar degeneration, J Neurology, 257:1406-08, 2010.

Shaikh, A, Wong, AL, Optican, LM, Miura, K, Solomon, D, **Zee,** DS, Sustained eye closure slows saccades, Vis Research, 50:1665-75, 2010.

Tian, J. and **Zee**, DS, Context-specific saccadic adaptation in monkeys, Vision Res, 50:2403-10. 2010.

Hullar, TE, **Zee**, DS, Minor, LB, Evaluation of the patient with dizziness, Chapter 164, pp 2305-2207, in Cummings Otolaryngology Head & Neck Surgery, Fifth edition, editors, Flint, PW et al. Mosby Elsevier, Philadelphia, 2010.

Crane, BT, Eggers, SDZ, **Zee**, DS, Baloh, RW, Central Vestibular Disorders, Chapter 166, 2346-2358, in Cummings Otolaryngology Head & Neck Surgery, Fifth edition, editors, Flint, PW et al. Mosby Elsevier, Philadelphia, 2010.

**2011**

Walker, M, Tian, J, Shan, X, Tamargo, R, Ying, H, **Zee,** DS, The cerebellar nodulus/uvula integrates otolith signals for the translational vestibulo-ocular reflex, PLoS ONE 5(11): e13981. doi:10.1371/ 2011.

Sotirchos, ES, Dorsey, ER, Tan, IL, **Zee,** DS, Opsoclonus-myoclonus syndrome and exaggerated startle response associated with small-cell lung cancer. Mov Disorders, 26:1768-69, 2011.

Shaikh, AG, Marti, S., Tartnutzer, A., Palla, A., Crawford, TO, Straumann, D., Carey, JP, Nguyen, KD, **Zee,** DS, Ataxia-telangiectasia: a “disease model” to understand cerebellar control of vestibular reflexes, J Neurophysiology, 105: 3034-41, 2011.

Shaikh, A, Xu-Wilson, M, Grill, S, **Zee,** DS**, ‘**Staircase’ square-waver jerks in early Parkinson’s disease, B J Ophthalmology, 95:705-09, 2011.

Oishi, K, Huang H, Yoshioka, T, Ying, S, **Zee,** DS, Zilles, K, Amunts, K, Woods, R, Toga, A, Pike, GB, Rosa-Neto, P, Evans, A, van Zijl, PCM., Mazziotta, JC, and Mori, S, Superficially located white natter structures commonly seen in the Macaque and the human brain with diffusion tensor imaging, Brain Connectivity, 1:37-48, 2011.

Strupp, M, Thurtell, MJ, Shaikh, AG, Brandt, T, **Zee**, DS, Leigh, RJ. Pharmacotherapy of vestibular and oculomotor disorders including nystagmus, J. Neurol 288; 1207-22 2011.

Olasagasti, I., Bockisch, C., **Zee,** DS, Straumann, D. Dynamic cyclovergence during vertical translation in humans, J Neuroscience, 31:9991-7, 2011.

Hong, S, Optican, LM, FitzGibbon, EJ, **Zee,** DS, Shaikh, AG, Eye movement visualization software, J Neuroscience Methods, 200: 181-184, 2011.

Shan, X, Hamasaki, I, Tian, J, Ying, H, Tamargo, R, **Zee,** DS. Vertical alignment in monkeys with unilateral IV section: effects of prolonged monocular patching and trigeminal deafferentation, Ann NY Acad Sci , 1233:78-84, 2011.

Xu-Wilson, M, Tian, J., Shadmehr, R, **Zee**, DS, TMS perturbs saccade trajectories and unmasks an internal feedback controller for saccades, J Neuroscience, 31:11537-46, 2011.

Wan, J, Mamsa, H, Johnston, JL, Spriggs, EL, Singer, HS, **Zee**, DS, Al-Bayati, AR, Baloh, RW, Jen, JC, Large Genomic Deletions in CACNA1A Cause Episodic Ataxia Type 2, Front.Neur. **2**:51. doi: 10.3389/fneur.2011.00051, 2011.

Kheradmand, A, **Zee,** DS, Cerebellum and Ocular Motor Control, Front.Neur. **2**:53. doi: 10.3389/fneur.2011.00053, 2011.

Roberts, DC, Marcelli, V, Gillen, JS, Carey, JP, DellaSantina, CC, **Zee**, DS, MRI magnetic field stimulates rotational sensors of the brain, Current Biology, 21:1635-40, 2011, (with an accompanying editorial in Current Biology and subsequently highlighted in Nature (2011), J Neurophysiology (2012) and Annals Neurology (2012)).

Lopez, J, **Zee** DS, Levy, L, Eye closure and oculopalatal tremor, Neurology, 77:1929, 2011.

Rucker J and **Zee** DS, editors, “Basic and Clinical Ocular Motor and Vestibular Research, Ann NY Acad Sci, vol. 1233, Wiley Blackwell, 2011.

**2012**

Tarnutzer, AA, Fernando, DP, Kheradmand, A, Lasker, AG, **Zee**, DS, Temporal constancy of perceived direction of gravity assessed by visual line adjustments, J Vest Res, 22:41-54, 2012.

**Zee**, DS, What the future holds for the study of saccades, Biocybernetics and Biomedical Engineering (BBE), 32:65-76, 2012.

Sharrief, AZ, Raffel, J, **Zee**, DS, Basal ganglia abnormalities in B12 deficiency mimicking methylmalonic acidemia, Arch Neurology, 69:769-772, 2012.

Jung, BC _,_ Choi, SI, Du, AX, Cuzzocreo, JL, Ying, HS, Landman, BA, Perlman, SL, Baloh, RW, **Zee,** DS, Toga, AW, Prince, JL, Ying, SH, MRI shows a region-specific pattern of atrophy in spinocerebellar ataxia type 2. Cerebellum, 11: 272-279, 2012.

Schubert, M, Migliaccio, A, Ng, TWC, Shaikh, AG, **Zee**, DS, The under-compensatory roll aVOR does not affect dynamic visual acuity, JARO, 13:517-525, 2012.

Salsano, E, Umeh, C, Rufa, A, Pareyson, D, **Zee**, DS, Vertical supranuclear gaze palsy in Niemann-Pick Type C disease, Neurological Sciences, 33:1225-32, 2012.

Kheradmand, A, **Zee**, DS, The bedside examination of the vestibulo-ocular reflex (VOR): An update, Revue Neurologique, 168:710-19, 2012.

Tarnutzer, AA, Fernando, DP, Lasker, AG, **Zee**, DS, How stable is perceived direction of gravity over extended periods in darkness, Exp Brain Res, 222:427-46, 2012.

Baloh, RW, Halmagyi, GM, **Zee**, DS: The history and future of neurootology, chapter in Neuro-otology, editor Kerber, K, Continuum Lifelong Learning Neuro;18, 1001–1015, 2012.

Grill, E, Bronstein, A, Furman, J, **Zee**, DS, Muller, M, International classification of functioning, disability and health (ICF) core set for patients with vertigo and dizziness, J Vestibular Res, 22: 261-271, 2012.

**2013**

Davalos-Bichara, M, Lin, FR, Carey, JP, Walston, JD, Fairman, JE, Schubert, MC, Barron, JS, Hughes, J, Millar, J, Spar, A, Weber, KL, Ying, HS, Zackowski, KM, **Zee**, D, Agarwal, Y, Development and validation of a falls grading scale, J Geriatr Phys Ther, 36:63-67, 2013.

Shaikh, A, Optican, LM, **Zee**, DS, Membrane mechanisms of tremor, chapter 2 in Contemporary Clinical Neuroscience, eds. Mario Manto and Guliana Gallimardi, pp 11-35, Springer, 2013.

Shaikh, A, Palla, A, Marti, S, Olasagasti, I, Optican, LM, **Zee**, DS, Straumann, D, Role of cerebellum in motion perception and vestibulo-ocular reflex – similarities and disparities, The Cerebellum,12:97-107, 2013.

Nuti, D, **Zee**, DS, Positional vertigo and benign paroxysmal positional vertigo, chapter 20 in Bronstein, A, Oxford Textbook of Vertigo and Imbalance, pp. 217-230, Oxford University Press, 2013.

Kheradmand, A, Bronstein, A, **Zee** DS, Clinical Bedside Examination, chapter 12 in Bronstein, A, Oxford Textbook of Vertigo and Imbalance, pp. 123-136, Oxford University Press, 2013.

Newman-Toker, D, Tehrani, AS, Mantokoudis, G, Pula, J, Guede, C, Kerber, K, Blitz, A, Ying, S, Hsieh, Y-H, Rothman, R, Hanley, D, **Zee**, DS, Kattah, JC, Quantitative video-oculography to help diagnose stroke in acute vertigo and dizziness: towards an "ECG" for the eyes, Stroke, 44:1158-61, 2013.

Park, H-K, Kim, J-S, Strupp, M, **Zee,** DS, Isolated floccular infarction: impaired vestibular responses to horizontal head impulse, J Neurology, 260:1576-82, 2013.

Antoniades, C, Ettinger, U, Gaymard, B, Gilchrist, I, Kristjánsson, A, Kennard, C, Leigh, RJ, Noorani, I, Pouget, P,

Smyrnis, N, Tarnowski, A, **Zee**, DS, Carpenter, RHS, An internationally standardised antisaccade protocol for clinical use, Vision Res, 84:1-5, 2013.

Shaikh, A, **Zee,** DS, Mandir, AS, Lederman, HM, and Crawford, TC, Disorders of upper limb movements in ataxia-telangiectasia, PLoS ONE, 8(6), e67042 2013.

Shaikh, A, Wong, A, **Zee**, DS, Jinnah, H, Keeping your head on target, J Neuroscience, 33:11281-95, 2013.

Tian, J, Ying, H, **Zee**, DS, Revisiting corrective saccades: role of visual feedback, Vision Res, 89:54-64, 2013.

Tarnutzer, A, Lasker, A, **Zee** DS, Continuous theta-burst stimulation of the right superior temporal gyrus impairs self-motion perception, Exp Brain Res, 230: 359-370, 2013.

Shaikh, A, Marti, S, Tarnutzer, AA, Crawford, TO, **Zee**, DS, Straumann, D, Effects of 4-aminopyridine on nystagmus and vestibulo-ocular reflex in ataxia-telangiectasia, J Neurology, 260:2728 – 2735, 2013

Newman-Toker, D, Kerber, KA, Hsieh, YH, Pula, JH, Omron, R, Saber Tehrnani, AS, Mantokoudis, G, Hanley, DF, **Zee**, DS, Kattah, JC, HINTS outperforms ABCD2 to screen for stroke in acute continuous vertigo and dizziness, Acad Emerg Med 20: 986-96, 2013.

**Zee**, DS, Shaikh, A, Neurology of eye movements: from control systems models to ion channels and genetics to targeted pharmacotherapy, editors Werner, JS and Chalupa, LM, The New Visual Neurosciences, MIT press, 997-990, 2013.

**2014**

Lee, S-H, Park, S-O, Kim, J-S, Kim, H-J, Yunusov, F, **Zee**, DS, Isolated unilateral infarction of the cerebellar tonsil: ocular motor findings. Ann Neurol 75:429-34, 2014.

Beh, SC, Tehrani, AS, Kheradmand, A, **Zee**, DS, Damping of monocular pendular nystagmus with vibration in a patient with multiple sclerosis. Neurology, online, DOI 10.1212/ 2014.

Kim, HG, Kim, J-S, Choi, JH, Choi, KD, **Zee** DS, Rebound upbeat nystagmus after lateral gaze in episodic ataxia The Cerebellum, published online January 14, 2014.

Wenzel, A, Ward, B, Schubert, M, Kheradmand, A, **Zee**, DS, Carey, JC, Patients with vestibular loss, Tullio phenomenon and pressure induced nystagmus: vestibular atelectasis? Otol. Neurotology, 35: 866-72. 2014.

Agrawal, Y., Schubert, MC, Migliaccio, AA, Schneider, E., **Zee**, DS, Carey, JP. Evaluation of quantitative head impulse testing using search coils vs. video-oculography in older individuals, Otol Neurotology, 35:283-88, 2014.

Kim, J-S **Zee**, DS, Benign Paroxysmal Positional Vertigo, N. Engl. J. Med, 370: 1138-47, 2014.

Lee SH, Newman-Toker, DE, **Zee**, DS, Schubert, MC, Compensatory saccade differences between outward versus inward head impulses in chronic unilateral vestibular hypofunction, J Clinical Neuroscience, 21: 1744-1749, 2014.

Ward, BK, Roberts, DC, Della Santina, CC, Carey, JP, **Zee**, DS, Magnetic vestibular stimulation in subjects with unilateral labyrinthine disorders, Frontiers in Neuro-otology, Front. Neurol. doi: 10.3389/fneur.2014.00028, 2014.

Ward, BK, Tan, G X-J, Roberts, DC, Della Santina, CC, **Zee**, DS, Carey, JP, Strong static magnetic fields elicit swimming behaviors consistent with direct vestibular stimulation in adult zebra fish, PLoS ONE 9(3): e92109. doi:10.1371/journal.pone. 0092109, 2014.

Saber Tehrnani, AS, Kattah, JC, Mantokoudis, G, Pula, JH, Nair, D, Blitz, AG, Ying, S, Hanley, D, Z**ee**, DS, Newman-Toker, D, Small strokes causing severe vertigo: Frequency of false-negative MRIs and non-lacunar mechanisms. Neurology, 83:169-73, 2014.

Mantokoudis G, Saber Tehrani AS, Kattah JC, Eibenberger K, Guede I, **Zee** DS, Newman-Toker DE. Quantifying the vestibulo-ocular reflex with video-oculography: nature and frequency of artifacts, Audiol Neurotol, 20:39-50, 2014.

Mandalà M, Ramat S, **Zee** DS, Professor Daniele Nuti and his academic career, J Vestib Res.24:327, 2014.

**2015**

Daye, PM, Roberts, DC, **Zee,** DS, Optican, LM, Vestibulo-ocular reflex suppression during head-fixed saccades reveals gaze feedback control, J. Neuroscience, 35:1192-98, 2015.

Ward, BK, Roberts, DC, Della Santina, CC, Carey, JP, **Zee**, DS, Vestibular stimulation by magnetic fields, Ann NY Acad Sci, 1343:69-79, 2015.

Beh, SC, Murthusamy, B, Calabresi, P, Hart, J, **Zee**, D, Patel, V, Frohman, E. Hiding in plain sight: a closer look at posterior cortical atrophy, Pract Neurol,15:5-13, 2015.

Kheradmand, A, Lasker, A. **Zee**, DS, Transcranial magnetic stimulation (TMS) of the supramarginal gyrus– A window to perception of upright, Cerebral Cortex, 25:765-71, 2015.

Huh, YE, Kim, J-S, Kim, H-J, Park, S-H, Jeon, BS, Kim, J-M, Chu, JW, **Zee** DS. Vestibular performance during high-acceleration stimuli correlates with clinical decline in SCA6, Cerebellum, 14:284-9, 2015.

Patel, VR, **Zee,** DS, The cerebellum in eye movement control: nystagmus, coordinate frames and disconjugacy, Eye, 29:191-195, 2015.

Shaikh, AG, Wong, A, **Zee,** DS, Jinnah, H, Why are voluntary head movements in cervical dystonia slow? Parkinsonism & Related Disorders, 21:561-6, 2015.

Shaikh, AG, **Zee,** DS, Jinnah, H, Oscillatory head movements in cervical dystonia: Dystonia, tremor, or both? Movement Disorders, 30:834-42, 2015.

Mantokoudis G1, Saber Tehrani AS, Wozniak A, Eibenberger K, Kattah JC, Guede CI, **Zee** DS, Newman-Toker DE, VOR gain by head impulse video-oculography differentiates acute vestibular neuritis from stroke, Otol Neurotol, 36:457-6, 2015.

Ward, BK, **Zee,** DS, Solomon, D, Gallia, GL, Reh, DD, Cerebrospinal fluid leak: a complication from vomiting after magnetic vestibular stimulation, Neurology, 85:551-2, 2015.

Leigh, RJ, **Zee**, DS, The Neurology of Eye Movements, Fifth edition, Oxford University Press, New York, 2015.

Otero-Millan, J, Benavides, D., **Zee**, DS, Kheradmand, A., Bilateral INO: Unusual patterns of saccadic intrusions, Neurology, 85:1428-1429, 2015.

Gold, DR, Zee, DS, Neuro-ophthalmology and Neuro-otology Update, J Neurology, 262:2786-2792, 2015.

Otero-Millan, J, Roberts, DC, Lasker, A, **Zee**, DS, Kheradmand, A. Knowing what the brain is seeing in three dimensions: a novel, noninvasive, sensitive, accurate, and low noise technique for measuring ocular torsion, J Vision, 15:11, 2015.

**2016**

Mantokoudis, G, Saber Tehrani, AS, Xie, L, Eibenberger, K, Eibenberger, B, Roberts, D, Newman-Toker, DE, **Zee**, DS, The video head impulse test during post-rotatory nystagmus: physiology and clinical implications, Exp Brain Res., 234:277-286, 2016.

Jareonsettasin, P, Otero-Millan, J, Ward, BK, Roberts, DA, Schubert, MC, **Zee**, DS, Sustained magnetic field stimulation of the labyrinth reveals multiple time constants of vestibular set-point (bias) adaptation, Curr Biology, 26:1359-65, 2016 (NIHMS788462) PMID: 27185559.

Umeh, C., Polydefikis, M., Chaudhry, V. and **Zee**, DS, Sweat gland denervation in cerebellar ataxia with neuropathy and vestibular areflexia syndrome, Movement Disorders, Clinical Practice, 4: 46-48, 2016.

Choi, K-D, Shin, HK, Kim, J-S, Kim, S-H, Choi, J-H, Kim, H-J, **Zee**, DS, Variants of windmill nystagmus, J Neurology, 263:1375-81, 2016.

Quiroga, AC, Sabatino, JJ, **Zee**, DS, Gold, DR, The hopping lid twitch in myasthenia gravis, Neurology,87:e55, 2016

Kheradmand, A, Colpak, AI, **Zee**, DS. Eye movements in vestibular disorders. Chapter 9, in Handb Clin Neurol.;137:103-17, 2016.

Gold, DR, **Zee,** DS, Dizziness in Seminars in Neurology: Pearls and Pitfalls 36: 433-441, 2016.

Shaikh, AG, **Zee**, DS, Crawford, JD, Jinnah, HA, Cervical dystonia: Disorder of a neural integrator, Brain, 139: 2590-2599, 2016.

Mantokoudis G1, Saber Tehrani AS, Wozniak A, Eibenberger K, Kattah JC, Guede CI, **Zee** DS, Newman-Toker DE, Impact of artifacts on VOR gain measures by video-oculography in the acute vestibular syndrome, J Vestib Res, 26: 375-385, 2016.

Kim, S-H, **Zee**, DS, du Lac, S, Kim, HJ, Kim, J-S, Nucleus prepositus hypoglossi lesions produce a unique ocular motor syndrome, Neurology, 87:2026-2033, 2016.

Ward, B and **Zee,** D, Dizziness and vertigo in MRI machines, N. Engl. J. Med, 375:e44, 2016.

Khehadmand, A, Kim, J-S, **Zee**, D, Cerebellum and Oculomotor Deficits, in Essentials of Cerebellum and Cerebellar Disorders, editors Gruol, D et al., Springer, pp. 471-6, 2016.

**2017**

**Zee**, DS, Jareonsettasin, P, Leigh, RJ, Ocular stability and set-point adaptation, Phil. Trans. R. Soc. B, published online, 372:1718, 2017.

Otero-Millan J, Treviño, C, Winnick, A, **Zee**, DS, Carey, JP, Kheradmand, A, The video ocular counterroll (vOCR): A clinical test to detect loss of otolith-ocular function, Acta Otolaryngol, 137:593-597, 2017.

Lee, SU, Choi, JY, Kim, HJ, Park JJ, **Zee,** DS, Kim, J-S, [Impaired tilt suppression of post-rotatory nystagmus and cross-coupled head-shaking nystagmus in cerebellar lesions: image mapping study](http://www.ncbi.nlm.nih.gov/pubmed/26969184), The Cerebellum, 16:95-102, 2017

Kass, BM, **Zee,** DS, Kheradmand, A, Gold, DR, Midbrain infarction resulting in bilateral pseudoabducens palsies, The Neurologist, 22:72-76, 2017.

Shaikh, AG, **Zee**, DS, Crawford, JD, Jinnah, HA, Reply: Contributions of visual and motor signals in cervical dystonia, Letter, Brain, 140: published online, 2017.

Otero-Millan, J, **Zee**, DS, Schubert, MD, Roberts, DC, Ward, BK, Three-dimensional eye movement recordings during magnetic vestibular stimulation, J. Neurology, published online, 2017

Shaikh, AG, Wong, AL, Optican, LM, and **Zee**, DS, Impaired motor learning in a disorder of the inferior olive: Is the cerebellum confused? The Cerebellum, 16:158-167, 2017.

Ward, BK, Otero-Millan, J, Jareonsettasin, P, Schubert, MC, Roberts, DC, **Zee**, DS, Magnetic vestibular stimulation (MVS) as a technique for understanding the normal and diseased labyrinth, Frontiers in Neurology, published online, 2017.

Colagiorgio, P, Versino, M, Colnaghi, S, Quaglieri, S, Manfrin, M, Zamaro, E, Mantokoudis, G, **Zee** DS, and Ramat, S, New insights into vestibular-saccade interaction based on covert corrective saccades in patients with unilateral vestibular deficits, J. Neurophysiol., 117:2324-2338, 2017.

Schiess, N, **Zee**, DS, Siddiqui, KA, Szolics M, El Hattab, AW, Novel PNKP Mutation in siblings with Ataxia-Oculomotor Apraxia Type 4, J. Neurogenetics, 31: 23-25, 2017.

Yacovino, DA, Akly, MP, Leonel, L, **Zee**, DS, The floccular syndrome: dynamic changes in eye movements and vestibulo-ocular reflex in isolated infarction of the cerebellar flocculus, The Cerebellum, published online, 2017.

Oh, EH, Lee , J-H, Shin, J-H, Kim, H-S, Kim, J-S, Kim, H-J, Choi, S-Y, Choi, K-D, **Zee**, DS, Cho, J-H, Patterns and modulations of Pendular nystagmus in a family with hereditary spastic paraplegia, J Neurological Sci. 383: 169-173, 2017.

Shaikh, AG and **Zee,** DS, Eye Movement Research in the Twenty-First Century—a Window to the Brain, Mind, and More, The Cerebellum, published online, 2017.

**2018**

Kronenbuerger, M, Olivi, A, **Zee**, DS, A picture is worth a thousand words: Positional vertigo and vertical nystagmus in medulloblastoma, Neurology, in press, 2018.

Tehrani, AS, Kattah, J, Kerber, K, Gold, D, **Zee** DS, Urrutia, V, Newman-Toker, D, Diagnosing stroke in acute dizziness and vertigo: Pitfalls and Pearls, Stroke, in press, 2018.

Choi, J-Y, Glasauer, S, Choi, SY, Kim, JH, **Zee**, DS, Kim, J-S, Apogeotropic central positional nystagmus: Characteristics and mechanism, Brain, in press, 2018.
